# Polymorphisms in genes expressed during amelogenesis and their association with dental caries: a case–control study

**DOI:** 10.1007/s00784-022-04794-2

**Published:** 2022-11-24

**Authors:** Daniela Gachova, Bretislav Lipovy, Tereza Deissova, Lydie Izakovicova Holla, Zdenek Danek, Petra Borilova Linhartova

**Affiliations:** 1 RECETOX, Faculty of Science, Masaryk University, Kotlarska 2, Brno, Czech Republic; 2 Department of Burns and Plastic Surgery, Faculty of Medicine, Institution Shared With the University Hospital Brno, Masaryk University, Jihlavska 20, 62500 Brno, Czech Republic; 3 Clinic of Stomatology, Faculty of Medicine, Institution Shared With St. Anne’s University Hospital, Masaryk University, Pekarska 664/53, 60200 Brno, Czech Republic; 4 Clinic of Maxillofacial Surgery, Faculty of Medicine, Institution Shared With the University Hospital Brno, Masaryk University, Jihlavska 20, 62500 Brno, Czech Republic; 5 Department of Pathophysiology, Faculty of Medicine, Masaryk University, Kamenice 5, 62500 Brno, Czech Republic

**Keywords:** Dental caries, Tooth morphology, Amelogenin, Kallikrein 4, Gene polymorphism

## Abstract

**Objectives:**

Dental caries is a widespread multifactorial disease, caused by the demineralization of hard dental tissues. Susceptibility to dental caries is partially genetically conditioned; this study was aimed at finding an association of selected single nucleotide polymorphisms (SNPs) in genes encoding proteins involved in amelogenesis with this disease in children.

**Materials and methods:**

In this case–control study, 15 SNPs in *ALOX15*, *AMBN*, *AMELX*, *KLK4*, *TFIP11*, and *TUFT1* genes were analyzed in 150 children with primary dentition and 611 children with permanent teeth with/without dental caries from the European Longitudinal Study of Pregnancy and Childhood (ELSPAC) cohort.

**Results:**

Dental caries in primary dentition was associated with SNPs in *AMELX* (rs17878486) and *KLK4* (rs198968, rs2242670), and dental caries in permanent dentition with SNPs in *AMELX* (rs17878486) and *KLK4* (rs2235091, rs2242670, rs2978642), (*p* ≤ 0.05). No significant differences between cases and controls were observed in the allele or genotype frequencies of any of the selected SNPs in *ALOX15*, *AMBN*, *TFIP11*, and *TUFT1* genes (*p* > 0.05). Some *KLK4* haplotypes were associated with dental caries in permanent dentition (*p* ≤ 0.05).

**Conclusions:**

Based on this study, we found that although the SNPs in *AMELX* and *KLK4* are localized in intronic regions and their functional significance has not yet been determined, they are associated with susceptibility to dental caries in children.

**Clinical relevance:**

*AMELX* and *KLK4* variants could be considered in the risk assessment of dental caries, especially in permanent dentition, in the European Caucasian population.

**Supplementary Information:**

The online version contains supplementary material available at 10.1007/s00784-022-04794-2.

## Introduction

Dental caries is caused by demineralization of the hard tissues of the tooth (enamel and dentine), which subsequently allows it to progress, and can lead to pulpitis and apical periodontitis. The term “early childhood caries” (ECC) is defined as the presence of at least one carious tooth, filled tooth, or tooth extracted as a result of caries in the dentition of a 6-year-old or younger child [1]. Previous studies reported that the presence of ECC influences also the occurrence of dental caries during adolescence [2, 3].

As the morphology of the primary dentition differs from that of permanent dentition, there are differences also in the degree of mineralization between these two types of teeth (75% in primary compared to 90% in permanent dentition, respectively). In primary teeth, the process of demineralization is faster than in permanent teeth and the rate of progression differs between these types of dentition [4].

The development of dental caries can be affected by a wide range of internal and external factors, including dental hygiene (the amount of dental plaque), dietary habits (the supply of fermentable saccharides), application of fluoride, the amount and composition of saliva, and genetic predispositions. Variability in multiple genes has been investigated in association with dental caries, such as genes affecting food/taste preferences, immune response, or genes encoding proteins involved in the enamel formation (amelogenesis), and mineralization.

This study was aimed at identifying and analyzing possible associations between the variability in *ALOX15*, *AMBN*, *AMELX*, *KLK4*, *TFIP11*, and *TUFT1* genes (all of which are expressed during amelogenesis) and the occurrence of dental caries in the primary and permanent dentition in Czech children (European Caucasian population).

## Materials and methods

### Clinical examination and sample collection

This case–control genetic association study conducted from 2005 to 2020 included 761 children from the South Moravia region of the Czech Republic (European Caucasian population). Participating children were classified based on their dentition into two groups with either solely primary or permanent dentition, with the latter group recruited within the scope of the European Longitudinal Study of Pregnancy and Childhood (ELSPAC) [5].

Inclusion and exclusion criteria for both groups of patients as well as clinical examinations were described in detail in our previous work [6]. The inclusion criteria for the group of children with primary dentition were at least 16 primary teeth, general good health, and the willingness of the parents to enroll their children in the study. The exclusion criteria for them were the presence of one or more permanent teeth, a familial relationship between children, and an ethnicity other than Czech Caucasian. The inclusion criteria for group of children with permanent dentition were age 13–15 years, general good health, and the willingness of the parents to enroll their children in the study [7]. The exclusion criteria for this group were previous or current therapy with orthodontic appliances, a familial relationship between children, and ethnicity other than Czech Caucasian.

The group of children with primary dentition were recruited from the outpatient clinic at the Department of Paediatric Dentistry, University Hospital Brno, and the Paediatric Department of the Clinic of Stomatology, St. Anne’s Faculty Hospital in Brno. Patients underwent a preventive examination (healthy children) or complex treatment under general anaesthesia (children who were unable to undergo standard dental treatment due to their uncooperativeness and the need for multiple restorations and/or extractions) during 2016–2018. Oral examinations were performed by two mutually calibrated experienced pediatric dentists under standard conditions (using a dental probe and mirror after drying in good lighting conditions) in a professional dental unit. The decayed-missing-filled teeth (DMFT) index was calculated using dental caries (D3 level) as a cut-off point for the detection of decay [7].

The group of children with permanent dentition selected from the ELSPAC cohort underwent dental examinations at the Clinic of Stomatology, St. Anne’s University Hospital, in the period of 2005–2007. To determine the DMFT index for each child, the teeth were first dried and then examined in a good light; all existing teeth were examined in the dentist’s chair using a mirror and a dental probe. The data was entered into a standard medical record. A radiograph examination was not performed as it was not part of the routine dental care for these adolescents and would, therefore, be deemed unethical. The clinical assessment was carried out by one investigator using the cavitation of the lesion as the detection threshold of caries according to the criteria given in the WHO Oral Health Surveys. To minimize inter-examiner variability in the classification of carious lesions, the participating dentists were trained and calibrated in accurate dmft/DMFT charting.

Children with dmft/DMFT = 0 were considered controls, and children with dmft/DMFT > 0 were considered cases. All cases from the group with primary dentition could be classified as severe early childhood caries (sECC, defined as dmft ≥ 6 in children aged 6 or less), dmft in all such cases in our group ranged between 10 and 20; a subgroup of cases from the group with permanent dentition with DMFT ≥ 6 was also created to represent extreme cases of dental caries.

The study was approved by the Ethics Committee of the Faculty of Medicine, Masaryk University, Brno (3/2004, from March 30, 2004), and St. Anne’s University Hospital (no number assigned during original approval on April 13, 2004; Approval No. 1G/2017 assigned on June 24, 2016). Informed consent was obtained from all legal guardians of children, in line with the Helsinki Declaration, prior to their inclusion in the study.

Blood samples were taken from children with sECC; in all other participants, samples of buccal epithelial cells were collected. The DNA from swabs was isolated using the Ultra-Clean®BloodSpin® DNA Isolation Kit (Mo Bio Laboratories, Inc., Carlsbad, CA, USA) or according to the protocol reported previously [7]. Both approaches were compared and resulted in sufficient yields and purities.

### Genetic analysis

**Fig. 1:**
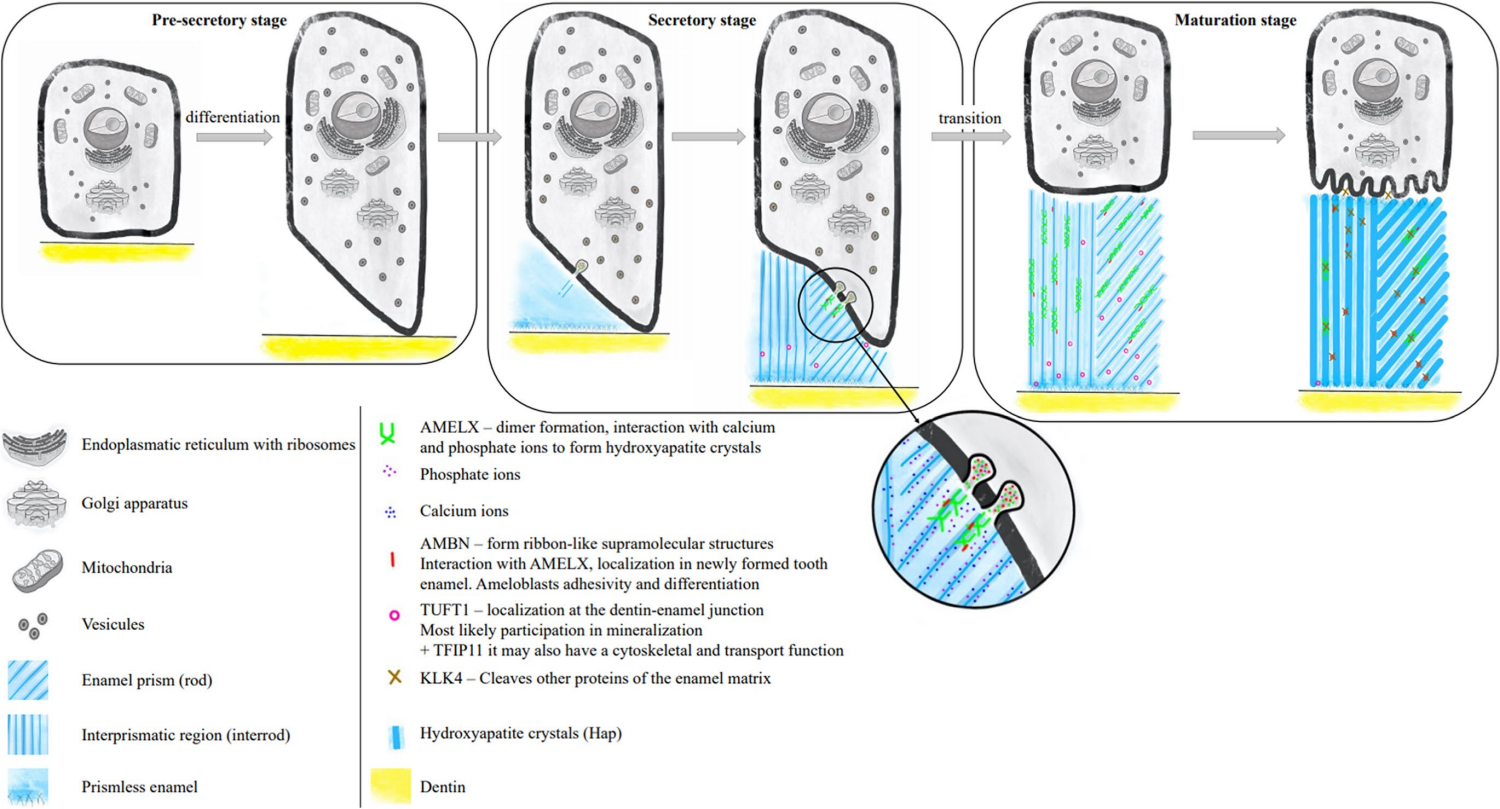
The process of amelogenesis — involvement of selected proteins in enamel formation and their function. During amelogenesis, ameloblasts go through various stages and perform various functions. During the pre-secretory phase, preameloblasts are formed from the enamel epithelium cells, initiate amelogenesis, and differentiate into secretory ameloblasts. The physiological differentiation of ameloblasts is controlled by ameloblastin (AMBN). Secretory ameloblasts are elongated and form Tomes’ process on the apical end (towards the dental root). There, proteins of the enamel matrix, as well as Ca^2+^ and PO_4_^3−^ ions, are released during the secretory phase of amelogenesis. These proteins, especially amelogenin (AMELX), catalyze the formation of hydroxyapatite crystals, which are deposited in prisms. Ameloblasts gradually move further from the dentin-enamel border and the prisms grow longer. After the enamel reaches a required density, ameloblasts grow shorter, the Tomes process recedes, and the number of the cellular organelles decreases (transition phase). A maturation phase follows, during which the enamel undergoes mineralization. In that stage, the protein tuftelin 1 (TUFT1) plays an important role and interacts with the tuftelin-interacting protein 11 (TFIP11). During the maturation stage, ameloblasts oscillate between ruffled (80%) and smooth-ended (20%) phases. In Fig. 1, only ruffle-ended ameloblasts are visualized. Proteases, such as kallikrein 4 (KLK4), are secreted and break down the enamel matrix proteins. Additional hydroxyapatite (Ca_10_(PO_4_)_6_(OH)_2_) crystals are being deposited into the prisms that grow in width until all gaps are filled. Once the tooth erupts, ameloblasts disappear and the enamel cannot, therefore, be renewed if the tooth is damaged [8–14]

Genes encoding proteins involved in amelogenesis, such as ameloblastin (*AMBN*), amelogenin (*AMELX*), kallikrein 4 (*KLK4*), tuftelin-interacting protein 11 (*TFIP11*), and tuftelin 1 (*TUFT1*), are considered candidate genes for the development of dental caries. The process of amelogenesis is summarized in Fig. 1. Arachidonate 15-lipoxygenase (ALOX15) has not been directly associated with amelogenesis so far; nevertheless, it is involved in bone mineralization, and it is, therefore, reasonable to assume that it can be involved in the formation of dental hard tissues as well. An overview of single nucleotide polymorphisms (SNPs) in these candidate genes, the association of which with dental caries in the primary and/or permanent dentition has been studied in various populations, is provided in Table S1 (Online Resource).

SNPs in *ALOX15* (rs2619112, rs7217186), *AMBN* (rs34538475), *AMELX* (rs946252, rs17878486), *KLK4* (rs198968, rs2235091, rs2242670, rs2978642), *TFIP11* (rs134136, rs5997096), and *TUFT1* (rs2337359, rs2337360, rs3790506, rs4970957) were selected for this study based on (i) associations with enamel defects and/dental caries described previously in other populations (Table S1, Online Resource), (ii) proven functional effects, (iii) known expression in amelogenesis, and (iv) minor allele frequency (MAF) higher than 0.1 in the studied population (Table 1).

**Table 1:** Minor allele frequencies (MAF) in the European population (EUR) and Czech population (CZ) of 15 selected single nucleotide polymorphisms (according to the NCBI, 2021 and the current study, 2022)

Gene	SNP	Alleles	MAF in EUR population (1000 genomes)	MAF in CZ population (194 subjects from the current study)	*p* value	Cramer’s *V* (Φ_C_) coefficient
*ALOX15*	rs2619112	A/G	A = 44.4%	A = 49.1%*	0.102	0.034
	rs7217186	C/T	C = 45.6%	C = 49.0%	0.222	0.025
*AMBN*	rs34538475	G/T	T = 23.9%	T = 25.8%	0.438	0.016
*AMELX*	rs946252	C/T	T = 33.4%	T = 25.1%*	**0.002**	**0.063**
	rs17878486	C/T	C = 25.2%	C = 26.5%	0.567	0.011
*KLK4*	rs198968	A/G	A = 16.6%	A = 19.1%	0.238	0.024
	rs2235091	G/A	G = 39.4%	G = 38.9%	0.865	0.004
	rs2242670	A/G	A = 45.2%	A = 49.0%	0.182	0.028
	rs2978642	A/T	T = 25.0%	T = 29.9%	**0.049**	**0.041**
*TFIP11*	rs134136	C/T	T = 34.6%	T = 41.5%	**0.011**	**0.053**
	rs5997096	C/T	T = 44.4%	T = 49.5%	0.066	0.038
*TUFT1*	rs2337359	C/T	C = 17.5%	C = 21.4%	0.072	0.037
	rs2337360	A/G	A = 36.5%	A = 44.1%	**0.005**	**0.058**
	rs3790506	A/G	A = 26.1%	A = 28.9%	0.259	0.023
	rs4970957	A/G	G = 18.9%	G = 16.2%	0.225	0.025

Bold entries represents significant results

*ALOX15* arachidonate 15-lipoxygenase, *AMBN* ameloblastin, *AMELX* amelogenin, *KLK4* kallikrein 4, *SNP* single nucleotide polymorphism, *TFIP11* tuftelin-interacting protein 11, *TUFT1*, tuftelin 1

^*^In the Czech population, the data come from the 173 healthy individuals

Differences in MAFs between the EUR population and CZ population were tested by the Fisher exact test and Cramer’s *V* coefficient (Φ_C_) was used (all Φ_C_ < 0.1, which indicates small effect size and/or weak differences between compared populations despite statistically significant results)

#### Real-time polymerase chain reaction (qPCR)

Genotyping of 15 SNPs in *ALOX15*, *AMBN*, *AMELX*, *KLK4*, *TFIP11*, or *TUFT1* genes was based on polymerase chain reaction using 5′ nuclease TaqMan® assays (Table S2, Online Resource). Reaction mixtures were prepared, and conditions set according to the manufacturer’s instructions (Thermo Fisher Scientific, Waltham, MA, USA), and fluorescence was measured using the ABI PRISM 7000 Sequence Detection System. SDS version 1.2.3 software was used to analyze real-time and endpoint fluorescence data. Genotyping was performed by investigators unaware of the phenotype; 10% of samples were analyzed in duplicate; positive and negative controls were used.

For SNPs in *ALOX15* (rs7217186) and *KLK4* (rs2242670), restriction fragment length polymorphism (RFLP)-PCR was proposed and performed in addition to qPCR.

#### RFLP-PCR ALOX15 (rs7217186)

For analysis of SNP in *ALOX15* (rs7217186), primers 5′-CTAATGCACTGAGCGGGCAGGAA-3′ (forward) and 5′-AGCCTCCAGAGGTCGGAACG-3′ (reverse) were used for PCR. The proposal of primers and restriction analysis protocol was based on the study by Zhang et al. [15]. The primers were, however, amended based on the current sequence available in the NCBI database (https://www.ncbi.nlm.nih.gov/snp/rs7217186). Amplification of DNA fragments containing the *ALOX15* (rs7217186) polymorphism was carried out in a reaction volume of 25.0 μL containing 100 ng of genomic DNA, 0.5 μM solution of each primer, 0.04 U/μL of Taq polymerase, and 1 × Taq buffer with (NH_4_)_4_SO_2_, 3 mM of MgCl_2_, and 2 mM dNTPs mix (Thermo Fischer Scientific, Waltham, MA, USA). Denaturation for 5 min at 94 °C was followed by 38 cycles of 94 °C for 40 s, 58 °C for 40 s, and 72 °C for 40 s in the thermocycler Sensoquest labcycler (Schoeller, Prague, Czech Republic). The last synthesis step was extended to 10 min at 72 °C, followed by cooling for 10 min at 10 °C.

The PCR products were then digested with the *Bsr*BI restriction enzyme. The restriction was performed in a volume of 21.0 μL consisting of 8.0 μL of the PCR product, 1 × CutSmart Buffer, and 0.5 U/μL of *Bsr*BI enzyme (New England Biolabs, Ipswich, MA, USA) and incubated overnight at 37 °C. The digested product (412 + 54 bp for CC, 466 + 412 + 54 bp for CT, and 466 bp for TT genotype, respectively) was visualized by electrophoresis with 2% agarose gel, GelRed nucleic acid stain, and a size standard of 50-bp GeneRuler Fermentas (Thermo Fischer Scientific, Waltham, MA, USA).

#### RFLP-PCR KLK4 (rs2242670)

The program Primer-BLAST (https://www.ncbi.nlm.nih.gov/tools/primer-blast/) was used to design the primers for the analysis of the SNP in *KLK4* (rs2242670)—namely, the 5′-CTGGGAGGAGGACGGAATGA-3′ (forward) and 5′-CCCACCCTCTTCGCTAAACA-3′ (reverse). The DNA amplicon with the *KLK4* (rs2242670) polymorphism was acquired by PCR in a reaction volume of 25.0 μL containing 100 ng of genomic DNA, 0.5 μM solution of each primer, 0.04 U/μL of Taq polymerase, and 1 × Taq buffer with (NH_4_)_2_SO_4_, 3 mM MgCl_2_, and 2 mM dNTPs mix (Thermo Fischer Scientific, Waltham, MA, USA). Denaturation for 5 min at 95 °C was followed by 34 cycles of 95 °C for 30 s, 56.5 °C for 45 s, and 72 °C for 45 s in the thermocycler Sensoquest labcycler (Schoeller, Prague, Czech Republic). The last synthesis step was extended to 5 min at 72 °C followed by cooling for 10 min at 10 °C.

The restriction enzyme Hpy188III was chosen according to NEBcutter V2.0 (http://nc2.neb.com/NEBcutter2/). The restriction was performed in a volume of 27.0 μL consisting of 15.0 μL of the PCR product, 1 × CutSmart Buffer, and 0.04 U/μL of *Hpy188*III enzyme (New England Biolabs, Ipswich, MA, USA) and incubated overnight at 37 °C. The digested products, 131 + 91 bp (GG), 222 + 131 + 91 bp (AG), and 222 bp (AA), were electrophoresed in 3% agarose gel and visualized by GelRed nucleic acid staining using a size standard of 50-bp GeneRuler Fermentas (Thermo Fischer Scientific, Waltham, MA, USA).

### Statistical analysis

Absolute and relative frequencies are used for the presentation of categorical variables, and means and standard deviations (SD) for numerical variables. The allele frequencies were derived based on the number of genotypes acquired in the above-described analyses and compared by the Fisher exact test. Hardy–Weinberg equilibrium (HWE) and differences in the genotypes were analyzed using the chi-square test. To measure how strongly our population differs in allele frequencies from the European (EUR) population, we added Cramer’s *V* coefficient (Φ_C_; the scale used for interpretation was as follows: *φ* < 0.3, small effect; 0.3 ≤ *φ* < 0.50, medium effect; *φ* ≥ 0.5, large effect). Odds ratio (OR), confidence intervals (CI), and *p* values were calculated. All statistical analyses were performed using the program package Statistica v. 13 (StatSoft Inc., Tulsa, Okla., USA) and IBM SPSS Statistics v. 26 (IBM Corporation, 2019).

Haplotype frequencies were calculated in the software SNPAnalyzer 2.0; we used an algorithm embedded directly into the standardized SW toolkit for the correction of multiple comparisons [16].

The relation of the linkage disequilibrium (LD) to the physical distance within haplotype blocks is assessed employing the mean value of the correlation coefficient (*R*^2^) and the mean value of *D*′ (a normalized measure of allelic association that is known to fluctuate upward when a small number of samples or rare alleles are examined) [17]. Various pairwise LD statistics, such as *R*^2^ or *D*′, can be visualized using triangular heat maps in which the color shading indicates the strength and distribution of the pairwise LDs [18–20].

## Results

In total, the group with primary dentition comprised 150 preschool children (76 boys and 74 girls aged 2–6 years) with complete dentition. They were divided into cases (dmft ≥ 10; *N* = 105; 58 boys and 47 girls) and controls (DMFT = 0; *N* = 45; 18 boys and 27 girls).

The group with permanent dentition comprised 611 children (325 boys and 286 girls aged 13–15 years). The group included children with caries experience (cases, DMFT > 0; *N* = 462; 245 boys and 217 girls) and caries-free children (controls, DMFT = 0; *N* = 149; 80 boys and 69 girls). In addition, a separate analysis was also performed for a subgroup of these 462 cases from the group with permanent dentition consisting solely of children with DMFT ≥ 6 (*N* = 108; 60 boys and 48 girls).

There were no differences in the sex distribution betweet cases and controls in any of the groups (primary or permanent dentition, *p* > 0.05). Children with intact primary dentition (dmft = 0) were older than children with ECC (dmft > 0; *p* = 0.009). There was no significant difference in age between cases and controls in the group with permanent dentition (*p* > 0.05).

**Fig. 2:**
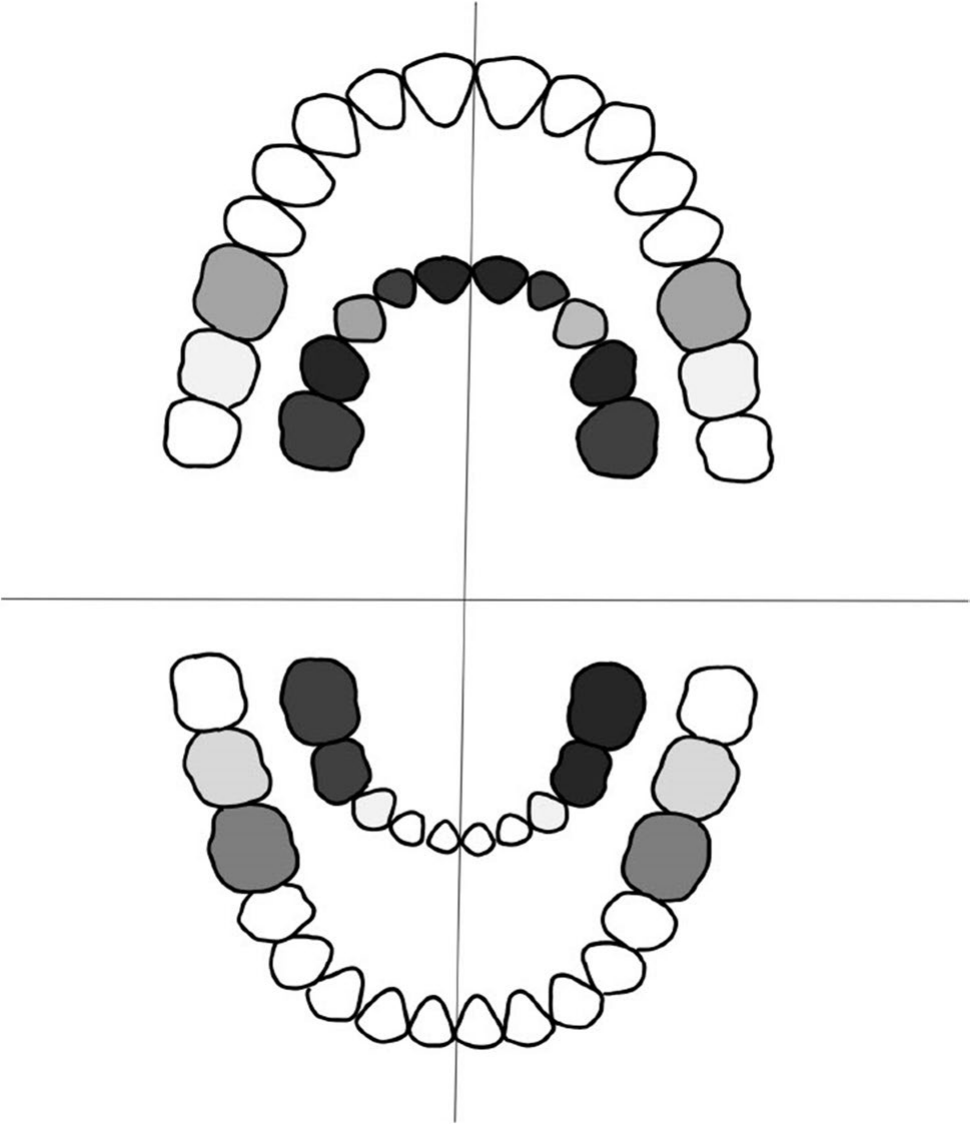
Teeth most frequently affected by dental caries in the studied population with dental caries. The inner scheme indicates the primary dentition (*N* = 103), and the outer permanent dentition (*N* = 462). The frequency of dental caries occurrence is indicated by the grayscale intensity

The lower incisors and canines (teeth 83, 82, 81, 71, 72, and 73 using the FDI World Dental Federation numbering) were the least affected teeth in the primary dentition. On the other hand, the most affected teeth in the primary dentition were the upper incisors and canines (teeth 52, 51, 61, and 62) and all the molars (teeth 55, 54, 64, 65, 85, 84, 74, and 75). In permanent dentition, the first molars (i.e., teeth 16, 26, 36, and 46; see Fig. 2) were the most commonly affected.

MAFs of the most commonly studied SNPs in the Czech population were comparable to those reported for the European population according to the NCBI database (all Φ_C_ < 0.1, which indicates small effect size and/or weak differences between compared populations https://www.ncbi.nlm.nih.gov/snp/rs7217186#frequency_tab) (see Table 1).

The observed *ALOX15* (rs2619112, rs7217186), *AMBN* (rs34538475), *KLK4* (rs198968, rs2235091, rs2242670, rs2978642), *TFIP11* (rs134136, rs5997096), and *TUFT1* (rs2337359, rs2337360, rs3790506, rs4970957) genotype distributions in children with dmft/DMFT = 0 were in HWE (*p* > 0.05). On the other hand, the *AMELX* (rs946252, rs17878486) variants were in HW disequilibrium in both control groups (see Tables 2 and 3 and Tables S3 and S4) (Online Resources).

**Table 2:** Allele and genotype frequencies of selected single nucleotide polymorphisms (SNPs) in gene encoding amelogenin (*AMELX*) for dental caries in children with primary and with permanent dentition

Gene *AMELX* SNP		Primary dmft = 0 *N* = 45 (%)	Primary dmft ≥ 10 *N* = 105 (%)	*p* value	Permanent DMFT = 0 *N* = 149 (%)	Permanent DMFT > 0 *N* = 462 (%)	*p* value	Permanent DMFT ≥ 6 *N* = 108 (%)	*p* value
rs946252♦	T	16 (30.8)	45 (22.5)		71 (24.1)	251 (27.6)		55 (25.9)	
	C	36 (69.2)	155 (77.5)	0.145	223 (75.9)	657 (72.4)	0.135	157 (74.1)	0.360
	TT	5 (19.2)	17 (17.0)		24 (16.3)	81 (17.8)		20 (18.9)	
	CT	6 (23.1)	11 (11.0)	0.234	23 (15.6)	89 (19.6)	0.449	15 (14.2)	0.848
	CC	15 (57.7)	72 (72.0)		100 (68.0)	284 (62.6)		71 (67.0)	
rs17878486	C	15 (16.7)	61 (29.0)	**0.015**	88 (29.5)	210 (22.7)	**0.011**	50 (23.1)	0.065
	T	75 (83.3)	149 (71.0)		210 (70.5)	714 (77.3)		166 (76.9)	
	CC	4 (8.9)	21 (20.0)	0.188	32 (21.5)	70 (15.2)	0.162	18 (16.7)	0.416
	CT	7 (15.6)	19 (18.1)		24 (16.1)	70 (15.2)		14 (13.0)	
	TT	34 (75.6)	65 (61.9)		93 (62.4)	322 (69.7)		76 (70.4)	
	CC + CT	11 (24.4)	40 (38.1)	0.075	56 (37.6)	140 (30.3)	0.061	32 (29.6)	0.116
	CT + TT	41 (91.1)	84 (80.0)	0.072	117 (78.5)	392 (84.8)	**0.049**	90 (83.3)	0.212

Bold entries represents significant results

*dmft* or *DMFT* decay/missing/filled tooth; *N* (%), values represent numbers (%) of subjects

^♦^Five subjects in the group with primary dentition with dmft ≥ 10 and 19 subjects with dmft = 0 were not analyzed, 8 subjects in the group with permanent dentition with DMFT > 0, 2 subjects with DMFT ≥ 6, and 2 subjects in with DMFT = 0 were not analyzed

Differences in allele frequencies were tested by the Fisher exact test

**Table 3:** Allele and genotype frequencies of selected SNPs in the gene encoding kallikrein 4 (*KLK4*) for dental caries in children with primary dentition and with permanent dentition

Gene *KLK4* SNP		Primary dmft = 0 *N* = 45 (%)	Primary dmft ≥ 10 *N* = 105 (%)	*p* value	Permanent DMFT = 0 *N* = 149 (%)	Permanent DMFT > 0 *N* = 462 (%)	*p* value	Permanent DMFT ≥ 6 *N* = 108 (%)	*p* value
rs198968	A	23 (25.6)	34 (16.2)	**0.043**	51 (17.1)	147 (15.9)	0.341	33 (15.3)	0.333
	G	67 (74.4)	176 (83.8)		247 (82.9)	777 (84.1)		183 (84.7)	
	AA	1 (2.2)	3 (2.9)	0.057	4 (2.7)	11 (2.4)	0.884	3 (2.8)	0.790
	AG	21 (46.7)	28 (26.7)		43 (28.9)	125 (27.1)		27 (25.0)	
	GG	23 (51.1)	74 (70.5)		102 (68.5)	326 (70.6)		78 (72.2)	
	AA + AG	22 (48.9)	31 (29.5)	**0.019**	47 (31.5)	136 (29.4)	0.348	30 (27.8)	0.305
rs2235091	G	34 (37.8)	85 (40.5)	0.380	117 (39.3)	296 (32.0)	**0.014**	58 (26.9)	**0.002**
	A	56 (62.2)	125 (59.5)		181 (60.7)	628 (68.0)		158 (73.1)	
	GG	9 (20.0)	21 (20.0)	0.795	25 (16.8)	49 (10.6)	0.067	5 (4.6)	**0.006**
	AG	16 (35.6)	43 (41.0)		67 (45.0)	198 (42.9)		48 (44.4)	
	AA	20 (44.4)	41 (39.0)		57 (38.3)	215 (46.5)		55 (50.9)	
	GG + AG	25 (55.6)	64 (61.0)	0.330	92 (61.7)	247 (53.5)	**0.047**	53 (49.1)	**0.029**
	AG + AA	36 (80.0)	84 (80.0)	0.581	124 (83.2)	413 (89.4)	**0.034**	103 (95.4)	**0.002**
rs2242670	A	47 (52.2)	91 (43.3)	0.099	143 (48.0)	398 (43.1)	0.078	86 (39.8)	**0.040**
	G	43 (47.8)	119 (56.7)		155 (52.0)	526 (56.9)		130 (60.2)	
	AA	11 (24.4)	24 (22.9)	0.125	35 (23.5)	90 (19.5)	0.340	17 (15.7)	0.187
	AG	25 (55.6)	43 (41.0)		73 (49.0)	218 (47.2)		52 (48.1)	
	GG	9 (20.0)	38 (36.2)		41 (27.5)	154 (33.3)		39 (36.1)	
	AA + AG	36 (80.0)	67 (63.8)	**0.036**	108 (72.5)	308 (66.7)	0.110	69 (63.9)	0.092
rs2978642	T	19 (21.1)	53 (25.2)	0.270	97 (32.6)	230 (24.9)	**0.006**	45 (20.8)	**0.002**
	A	71 (78.9)	157 (74.8)		201 (67.4)	694 (75.1)		171 (79.2)	
	TT	0 (0.0)	5 (4.8)	0.329	15 (10.1)	25 (5.4)	**0.028**	2 (1.9)	**0.007**
	AT	19 (42.2)	43 (41.0)		67 (45.0)	180 (39.0)		41 (38.0)	
	AA	26 (57.8)	57 (54.3)		67 (45.0)	257 (55.6)		65 (60.2)	
	TT + AT	19 (42.2)	48 (45.7)	0.416	82 (55.0)	205 (44.4)	**0.015**	43 (39.8)	**0.011**
	AT + AA	45 (100.0)	100 (95.2)	0.163	134 (89.9)	437 (94.6)	**0.039**	106 (98.1)	**0.007**

Bold entries represents significant results

*dmft* or *DMFT* decay/missing/filled tooth; *N* (%), values represent numbers (%) of subjects; *SNP* single nucleotide polymorphism

### Arachidonate 15-lipoxygenase, ameloblastin, tuftelin-interacting protein 11, tuftelin 1

No significant differences between controls and cases in allele or genotype frequencies were observed in any of the selected SNPs in *ALOX15*, *AMBN*, *TFIP11*, and *TUFT1* genes (*p* > 0.05, Tables S3 and S4) in either of the groups.

### Amelogenin

Interestingly, the C allele of the SNP in *AMELX* (rs17878486) was associated with a higher risk of dental caries in primary dentition (*p* = 0.015, OR = 2.05, 95% CI 1.09–3.84), while the opposite effect was observed in permanent dentition (*p* = 0.011, OR = 0.70, 95% CI 0.51–0.94, Table 2).

### Kallikrein 4

The carriers of the G allele and GG genotype in the SNP in *KLK4* (rs198968) in the group with primary dentition were at a higher risk of dental caries compared to the carriers of the A allele and AA + AG genotypes, respectively (*p* = 0.043, OR = 1.78, 95% CI 0.98–3.24, and *p* = 0.019, OR = 2.28, 95% CI 1.11–4.69). On the other hand, the allele and genotype frequencies did not significantly differ between cases and controls in the group with permanent dentition (*p* > 0.05).

No significant differences in the allele and genotype distributions of the SNP in *KLK4* (rs2235091) were observed between cases and controls in the group with primary dentition (*p* > 0.05). However, in the group with permanent dentition, the A allele of the SNP in *KLK4* (rs2235091) was statistically significantly more common in children with dental caries (*p* = 0.014, OR = 1.37, 95% CI 1.05–1.80) and it was also associated with dental caries in the additive (*p* = 0.047) and dominant (*p* = 0.034) models. The same applied to carriers of this allele with multiple dental caries (DMFT ≥ 6) in permanent dentition—A allele was shown to be a risk factor for the development of dental caries (*p* = 0.002, OR = 1.76, 95% CI 1.20–2.58) in both additive (*p* = 0.029) and dominant (*p* = 0.002) models; the AA genotype was more common in controls (*p* = 0.006, OR = 4.15, 95% CI 1.54–11.23).

In the group with primary dentition, the GG genotype of SNP in *KLK4* (rs2242670) was, when compared with AA + AG genotypes, less common in controls than in cases (*p* = 0.036, OR = 2.27, 95% CI 0.99–5.21). The G allele of this polymorphism was also associated with the elevated risk of severe dental caries (children with DMFT ≥ 6) in permanent dentition (*p* = 0.040, OR = 1.39, 95% CI 0.98–1.99).

While the SNP in *KLK4* (rs2978642) was not associated with dental caries in primary dentition (*p* > 0.05), the A allele and AA genotype SNPs in *KLK4* (rs2978642) were associated with dental caries in children with permanent dentition (*p* = 0.006, OR = 1.46, 95% CI 1.10–1.94, and *p* = 0.015, OR = 1.53, 95% CI 1.06–2.22). The effect of the A allele in children with permanent dentition was also significant in the additive (*p* = 0.015) and dominant (*p* = 0.039) models. This effect was even stronger in children with DMFT ≥ 6 (*p* = 0.002, OR = 1.83, 95% CI 1.22–2.76, and *p* = 0.011, OR = 1.85, 95% CI 1.12–3.06), additive model (*p* = 0.011), and dominant model (*p* = 0.007), respectively (see Table 3).

### Haplotype analysis

Haplotype analysis was performed for SNPs in *ALOX15*, *AMELX*, *KLK4*, *TUFT1* and *TFIP11*. No significant differences in frequencies of any of the haplotypes in *ALOX15*, *AMELX*, *KLK4*, *TUFT1*, and *TFIP11* between cases and controls in the group with primary dentition were detected (*p* > 0.05) (see Table 4 and Tables S5–S8).

**Table 4:** Haplotype analysis of single nucleotide polymorphisms (SNPs) in the gene encoding kallikrein 4 (*KLK4)* and their association with dental caries in the primary dentition with dmft ≥ 10 and permanent dentition with DMFT > 0 and DMFT ≥ 6

rs198968	rs2978642	rs2242670	rs2235091	Primary dmft = 0 *N* = 45 (%)	Primary dmft ≥ 10 *N* = 105 (%)	OR	CI	*p* value	Permanent DMFT = 0 *N* = 149 (%)	Permanent DMFT > 0 *N* = 462 (%)	OR	CI	*p* value	Permanent DMFT ≥ 6 *N* = 108 (%)	OR	CI	*p* value
G	A	G	A	41.5%	41.8%	1.046	0.635–1.724	0.859	36.5%	45.3%	1.568	1.200–2.048	**0.001**	43.1%	1.864	1.307–2.659	< **0.001**
G	T	A	G	16.2%	15.1%	0.932	0.478–1.817	0.837	18.6%	16.2%	0.775	0.556–1.081	0.137	16.7%	0.690	0.431–1.104	0.118
A	A	A	A	15.9%	11.5%	0.571	0.275–1.189	0.141	12.9%	11.4%	0.833	0.562–1.236	0.369	12.3%	0.869	0.509–1.485	0.607
A	A	A	G	7.9%	5.7%	0.567	0.220–1.460	0.248	3.4%	4.1%	1.269	0.626–2.575	0.500	3.3%	0.965	0.361–2.576	0.943
G	A	A	G	5.2%	8.0%	2.016	0.662–6.134	0.189	6.9%	6.6%	0.985	0.569–1.705	0.956	6.3%	0.835	0.386–1.805	0.644
G	A	G	G	3.4%	5.1%	1.603	0.436–5.889	0.460	4.7%	2.1%	0.288	0.130–0.639	**0.003**	3.6%	0.414	0.133–1.287	0.104
G	T	G	G	3.4%	4.5%	1.450	0.389–5.398	0.568	5.7%	4.6%	0.735	0.403–1.342	0.326	4.1%	0.333	0.110–1.009	**0.033ˣ**
G	A	A	A	3.3%	2.0%	0.420	0.083–2.123	0.301	2.6%	3.2%	1.347	0.584–3.107	0.473	3.2%	1.599	0.571–4.478	0.371
A	A	G	G	1.7%	1.2%	0.856	0.077–9.560	0.900	0.0%	0.2%	0.000	0.000–0.000	-	-	-	-	-
G	T	A	A	1.5%	2.9%	2.618	0.311–22.063	0.326	3.6%	2.9%	0.747	0.338–1.649	0.479	2.7%	0.452	0.121–1.691	0.213
G	T	G	A	0.0%	1.5%	0.000	0.000–0.000	-	4.3%	2.9%	0.548	0.258–1.165	0.129	3.9%	0.745	0.271–2.048	0.564
A	A	G	A	0.0%	0.5%	0.000	0.000–0.000	-	0.5%	0.4%	0.644	0.058–7.131	0.727	0.6%	1.381	0.086–22.209	0.820
A	T	G	A	-	-	-	-	-	0.3%	0.2%	0.644	0.058–7.131	0.727	0.2%	0.000	0.000–0.000	-

Bold entries represents significant results

*CI* confidence interval, *dmft* or *DMFT* decay/missing/filled tooth, *OR* odds ratio

ˣNot significant after the Bonferroni approach

Haplotypes are ordered according to decreasing haplotype frequency in the healthy controls from the group with primary dentition

**Fig. 3:**
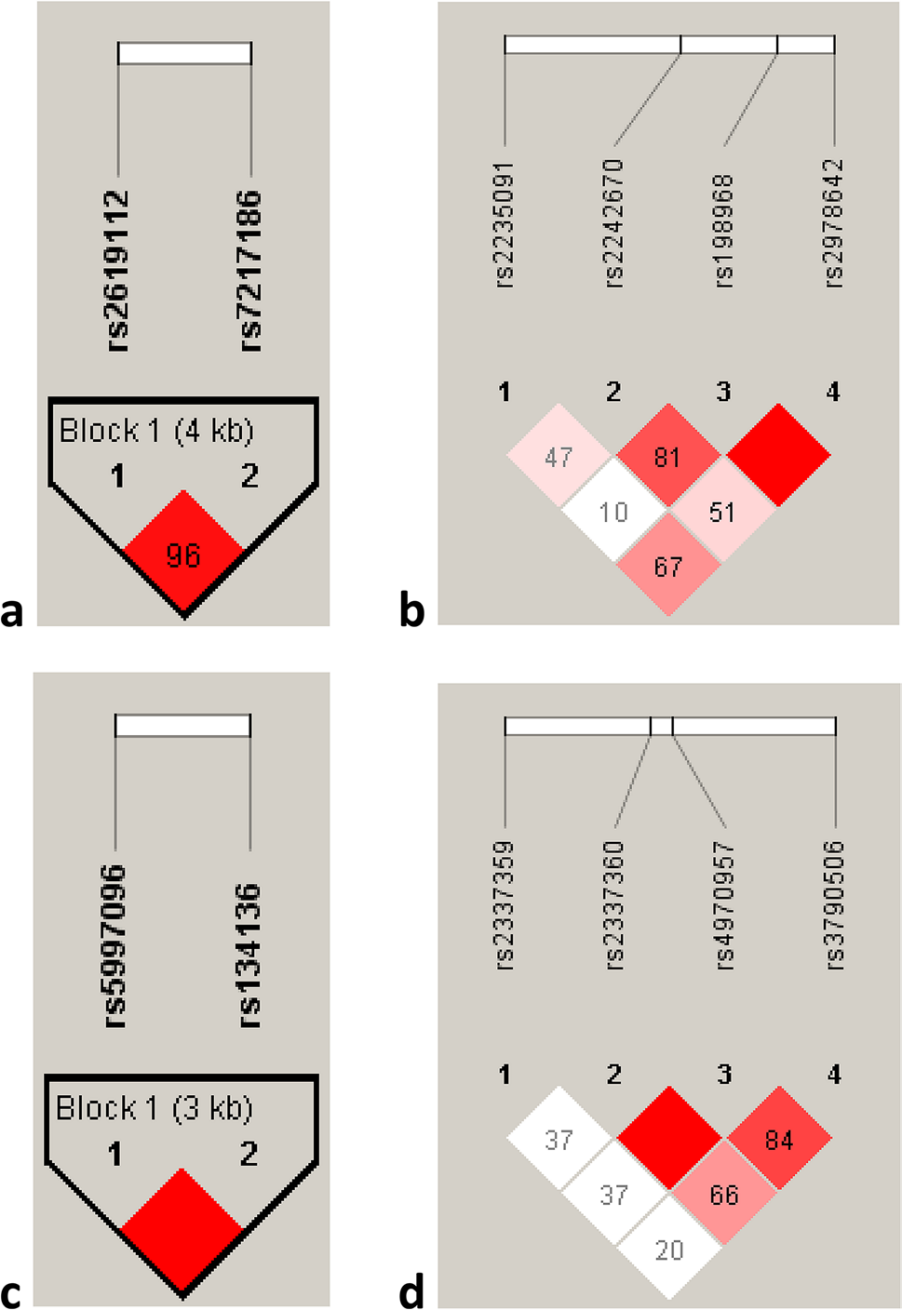
Linkage disequilibrium (LD) plots among the SNPs in controls in genes. **a** Arachidonate 15-lipoxygenase (*ALOX15*), **b** kallikrein 4 (*KLK4*), **c** tuftelin-interacting protein 11 (*TFIP11*), and **d** tuftelin 1 (*TUFT1*), respectively. *D*′ coefficient, strong LDs are highlighted in red. LD blocks (bold black line) were generated using the solid spine of the LD method

In children with permanent dentition, the haplotype AT in *ALOX15* (rs2619112/rs7217186) was less common in children with dental caries (DMFT > 0 and DMFT ≥ 6, Table S5, Online Resource) than in caries-free children (2.5% vs. 4.6%, *p* = 0.015, OR = 0.387, 95% CI = 0.184–0.814 for DMFT > 0 and 3.0% vs. 4.6%, *p* = 0.015, OR = 0.206, 95% CI = 0.046–0.922 for DMFT ≥ 6, respectively); these results, however, did not remain significant after Bonferroni correction (*p* > 0.05). A strong LD between SNPs rs2619112 and rs7217186 was observed (*D*′ = 96) in our study (see Fig. 3). The haplotype TC in *AMELX* (rs946252/rs17878486) was less common in children with dental caries (DMFT > 0, Table S6, Online Resource) than in controls with permanent dentition (0.9% vs. 2.0%, *p* = 0.046, OR = 0.256, 95% CI = 0.068–0.959), although this *p* value was not significant after Bonferroni correction (*p* > 0.05).

In the group with permanent dentition, the haplotype GAGA in *KLK4* (rs198968/rs2978642/rs2242670/rs2235091) was more common in children with dental caries (especially in children with DMFT ≥ 6) than in caries-free children (45.3% vs. 36.5%, *p* = 0.001, OR = 1.568, 95% CI = 1.200–2.048); on the other hand, the GAGG haplotype seems to be protective against dental caries (*p* = 0.003, OR = 0.288, 95% CI = 0.130–0.639, Table 4). The haplotype GTGG seems to be also protective against dental caries (*p* = 0.033, OR = 0.414, 95% CI = 0.133–1.287, Table 4); this, however, cannot be confirmed after Bonferroni correction (*p* > 0.05). LD plots between SNPs in the *KLK4* gene are shown in Fig. 3. A strong association was observed between *KLK4* rs198968 and rs2978642 (*D*′ =  ~ 100) as well as between rs198968 and rs2242670 (*D*′ = 81).

In patients with permanent dentition, the haplotype GCAA in *TUFT1* (rs3790506/rs2337359/rs2337360/rs4970957) was less common in cases than in controls for DMFT > 0 (1.9% vs. 4.9%, *p* = 0.022, OR = 0.191, 95% CI = 0.045–0.804, Table S8, Online Resource) and for DMFT ≥ 6 (3.0% vs. 4.9%, *p* = 0.014, OR = 0.205, CI = 0.046–0.918, Table S8, Online Resource). However, none of the *TUFT1* haplotypes showed any significant association with dental caries after Bonferroni correction (*p* > 0.05). Frequencies of all *TFIP11* haplotypes between cases (DMFT > 0) and controls (DMFT ≥ 6) were similar (*p* > 0.05, as calculated by a multiple model) (see Table S7) (Online Resource). LD plots among SNPs in *TFIP11* and *TUFT1* genes are shown in Fig. 3. LD plot based on *D*′ indicates a strong association (red in Fig. 3) between SNPs rs134136 and rs5997096 in the *TFIP11* gene. The *D′*-based LD plot also suggests a strong association between SNPs rs2237360 and rs4970957 in *TUFT1* and an intermediate association between rs4970957 and rs3790506 (*D*′ = 84).

## Discussion

In this study, we aimed to evaluate the associations between variants in certain genes expressed in amelogenesis as well as those previously associated with enamel defect and/or dental caries in the pediatric population. Some of such genes, such as the bone morphogenetic protein-2 (*BMP2*), distal-less homeobox 3 (*DLX3*) [21], enamelin (*ENAM*) [22], and matrix metalloproteinases (*MMPs*) [6] have been previously studied in the Czech population, although no associations between the selected variants in all candidate genes and the susceptibility to or severity of dental caries was found in neither primary nor permanent dentitions in children. For this reason, such genes were not included in this study.

In the studied cohort, the lower incisors and canines were the teeth least affected by dental caries. This can be explained by the fact that these teeth are washed by saliva from the sublingual salivary glands, which acts protectively against caries. Contrary, the upper incisors and canines are often affected by caries in young children because of their dietary habits, as indicated by the widely used terms such as “nursing caries” or “baby bottle syndrome” [23]. Among permanent teeth, the first molars were most commonly affected by dental caries. Caries in these teeth often occur on the occlusal or approximal surfaces, i.e., on habitually unclean surfaces.

### Arachidonate 15-lipoxygenase

ALOX15 is an oxidoreductase catalyzing the arachidonate oxidation, thus involved in the production of 15-hydroxyeicosatetraenoic acid participating in the regulation of anti-inflammatory response [24, 25]. ALOX15 also participates in the process of bone mineralization and its excessive amount can cause bone degradation [26, 27]. It is, therefore, possible that it affects the formation of hard dental tissues; nevertheless, its effect on the enamel structure or development of dental caries has not been fully clarified yet.

Abbasoğlu et al. [28] associated the SNP in *ALOX15* (rs7217186) with dental caries in primary dentition in Turkish children; the TT genotype was a risk factor for ECC. This is not in accordance with our results as this SNP has not been associated with dental caries in the Czech population in either of the groups (i.e., neither in the primary nor permanent dentition), although the MAFs are similar in the Czech and Turkish populations. In line with previous studies [28, 29], the other common SNP in this gene, *ALOX15* (rs2619112), was not associated with an increased risk of dental caries development in Czech children. Our study is the first one of variability in *ALOX15* about dental caries in the European population.

### Ameloblastin

A study in a mouse model by Fukumoto et al. [30] revealed that AMBN was important for proper ameloblast differentiation. This protein inhibits cell division and positively influences the adhesion of ameloblasts to the forming enamel [8, 31]. AMBN forms band-like supramolecular structures and interacts with AMELX [32–34], which supports the deposition of hydroxyapatite crystals into the prisms and interprismatic matrix. Mutations in this gene have been associated with amelogenesis imperfecta [35].

In our study, no association of the SNP in *AMBN* (rs34538475) with the development of dental caries was detected in either primary or permanent dentition in Czech children. This is in accordance with studies focusing on Turkish [28] or Guatemalan-Mayan [36] populations as well as with results of meta-analysis performed by Li et al. [37]. On the other hand, Gerreth et al. [38] associated this SNP with the occurrence of dental caries in primary dentition, with the T allele and TT genotype declared protective factors in the context of the dental caries development. However, the MAF in controls in the Polish population was 70.8% (*N* = 48), which is far from the mean MAF of the European population (EUR, 23.9%, NCBI, https://www.ncbi.nlm.nih.gov/snp/rs34538475#frequency_tab) as well as from our sample of the Czech population (MAF 25.8%, *N* = 194, Table 1). These differences in MAF in the studied populations could have led to this discrepancy of results (see Table S1 (Online Resource) for the full list of related studies).

### Amelogenin

According to the current knowledge, AMELX forms dimers and special structures interacting with Ca^2+^ and PO_4_^−^ ions, thus supporting the formation of hydroxyapatite and their storage in prisms [9, 39–41]. It is also assumed to have a buffering function [42]. The *AMELX* gene can be found on chromosome X; in males, a copy can be found on chromosome Y (*AMELY*). The gene contains 7 exons [43] that may undergo alternative mRNA splicing, which can, in turn, cause disorders in the enamel development [44]. Mutations in the *AMELX* gene are associated with the development of amelogenesis imperfecta [45–47].

In a meta-analysis that included 4 studies [38, 48–50], Sharifi et al. [51] did not prove any significant association between the SNPs in *AMELX* (rs946252 or rs17878486) and dental caries. Sensitivity analysis, however, indicated that *AMELX* (rs17878486) could be a risk factor for dental caries, which might have been hidden by other effective factors, namely, the ethnicity and type of selected controls. Some of the selected studies included even patients with dmft/DMFT ≤ 3 into the study, which represented a limitation of the meta-analysis, just like the failure to distinguish between the primary and permanent dentitions. In this context, it is important to note that in our study, the analysis of the SNP in *AMELX* (rs17878486) returned opposite results for permanent and primary dentitions. The C allele was associated with early childhood caries in the primary dentition, which is in agreement with the study by Patir et al. [52], but in opposition to the results reported by Gerreth et al. [38]. On the other hand, the T allele was shown to represent a risk for DMFT > 0 in permanent dentition, which was also confirmed when comparing genotypes (CC vs CT + TT), which is in agreement with results reported by Deeley et al. [36] in a Guatemalan-Mayan population. The association of the SNP in *AMELX* (rs17878486) with dental caries was confirmed also in a meta-analysis [37]. In their work, the C allele was associated with dental caries in an additive model; their study, however, did not distinguish between patients with primary and permanent dentition. The discrepancy in results can be caused by the differences in the design of individual studies as well as by the interpopulation variability (the studies differ in the age of individuals under study as well as other demographic aspects). According to the NCBI database (https://www.ncbi.nlm.nih.gov/snp/rs17878486#frequency_tab), the minority C allele of the SNP in *AMELX* (rs17878486) is present in 25.2% of the EUR population. The MAF in the Czech population is 26.5%, which is comparable with the frequency reported for the EUR population; in contrast, the study by Deeley et al. [36] claims that the T allele is the minority one (MAF 6%). It is, therefore, obvious that there is high variability in the frequencies of this SNP, which may be the reason for the discrepant results of individual studies. The SNP in *AMELX* (rs946252) itself was not associated with dental caries development in our study; however, the TC haplotype (SNPs rs946252 and rs17878486) was found to be marginally protective against the development of this disease in the permanent dentition.

### Kallikrein 4

KLK4 belongs to proteases, the main purpose of which is to replace the organic matter with minerals, thus contributing to the formation of the hard and less porous layer of enamel [53]. If the KLK4 function is impaired, so is the enamel maturation [8]; hydroxyapatite crystals fail to grow to the full width, and the prisms are not sufficiently interlinked as a large amount of the organic matrix remains between individual prisms [54]. Mutations in the *KLK4* gene were shown to be associated with the autosomal recessive form of amelogenesis imperfecta [55].

In our study, 4 SNPs in *KLK4* were analyzed, each of which was associated with the development of dental caries. In the primary dentition, the G allele of the SNP in *KLK4* (rs198968) was more frequently detected in patients with caries than in the controls with intact dentition. Similarly, the GG combination (compared to AA + AG genotypes) was associated with dental caries in the primary dentition, i.e., with ECC. In the Turkish population, Abbasoğlu et al. [28] found in a multivariate analysis considering environmental factors and gene-environment interactions that AG and GG genotypes are protective against ECC. This is not in agreement with our results; it is, however, necessary to point out the differences in MAF between the populations; while in the Czech Republic, the MAF of the SNP in *KLK4* (rs198968) was 19.1%, it was 31.3% in controls of the Turkish population [28].

The SNP in *KLK4* (rs2235091) was not associated with the occurrence of caries in the primary dentition in the Czech population, which is in agreement with the results from Turkey [28]. However, a study from Poland [38] on the SNP in *KLK4* (rs2235091) with a MAF of 34.0% and a study from USA [56] with a MAF of 38.0% reported the G allele to be a risk factor for caries in the primary dentition. The MAF in the Czech population was 38.9%, which is in accordance with the NCBI-reported MAF for EUR of 39.4%; the MAF did not differ significantly among populations in the aforementioned studies. Our results indicate that the A allele of this SNP is a risk factor for dental caries in the permanent dentition in the Czech population; moreover, the AA genotype was also associated with the severity of dental caries in the permanent dentition. Nevertheless, other studies did not show any association of this SNP with dental caries of permanent dentition [29, 57] or in mixed dentition [37]. The reasons for this difference may lie in the populations studied and/or in the design of the research. For example, Li et al. [37] considered patients with low caries experience as controls; other studied populations differed, for example, in the mean age of patients [29] or in ethnicity (9.5% of the population in Cavallari et al.’s paper [57] were Black).

Two more SNPs in *KLK4*, which were investigated in our study (rs2242670 and rs2978642), were also investigated by Cavallari et al. [57]. In their study, SNP in *KLK4* (rs2242670) was associated with dental caries in the permanent dentition; the AA genotype and combination of AA + AG genotypes were identified as risk factors. In our study, the GG genotype was associated with an increased risk of ECC and the A allele with dental caries in permanent dentition (DMFT ≥ 6). As far as the SNP in *KLK4* (rs2978642) is concerned, Cavallari et al. [57] found it to be borderline insignificant (*p* = 0.051) for the A allele (which indicates its possible association with the risk of dental caries) in the dominant model. Our results support those by Cavallari et al. [57]; however, the *p* values were more significant in our study (*p* = 0.006 to *p* = 0.039, Table 3), and the A allele, as well as the AA genotype and combined AA + AT vs. TT and AA vs. AT + TT, was risk-associated. In the primary dentition, SNPs in *KLK4* (rs2242670, rs2978642) were not associated with this disease in the Czech population, and, as it was the first study investigating their association with ECC, we cannot compare these results with the literature.

Two *KLK4* haplotypes were associated with caries in permanent dentition in the Czech population, even after Bonferroni correction. Namely, the haplotype GAGA (in the SNP order rs198968, rs2978642, rs2242670, and rs2235091; *p* = 0.001 for DMFT > 0 and *p* < 0.001 for DMFT ≥ 6) represented a pro-carious risk factor in permanent dentition and the GAGG haplotype (same order; *p* = 0.003 for DMFT > 0), which was in our population found to be protective against caries. Haplotype analysis showed that the SNP in *KLK4* (rs2235091) is the key factor for the resulting phenotype, in which the A allele is associated with increased risk while the G allele was protective. This corresponds also to the results of the comparison of the individual allele distribution between cases and controls in children with permanent dentition performed separately for this SNP. Our study is the only one published so far that simultaneously included all these 4 SNPs into a haplotype analysis. Cavallari et al. [57] studied SNPs rs2978642, rs2242670, and rs2235091 (and rs2978643), but they did not confirm the association with the disease.

### Tuftelin 1 and tuftelin-interacting protein 11

TUFT1 interacts with TFIP11; a complex of these two proteins was found at the apical end of ameloblasts and in the freshly formed enamel matrix. This localization and interaction suggest that TFIP11 might have a special cytoskeletal function and be involved in the transport or secretion of extracellular matrix proteins (AMELX), thereby influencing amelogenesis [58]. TUFT1 counts among non-amelogenins and is likely involved in the initial stage of enamel mineralization [59]. In a mouse model, Luo et al. [60] found out that excessive *TUFT1* expression leads to imperfect prism structure. Even though TUFT1 function has not been fully clarified yet, sequence changes in the *TUFT1* gene can interfere with other gene or protein interactions, thereby influencing, among other things, the development of dental caries [61].

Two SNPs in *TFIP11* (rs134136 and rs5997096) have been investigated in our study. No association with dental caries was found in any of these in the Czech population, neither in the primary nor permanent dentition. Other studies [28, 36–38, 52] corroborate our findings in both types of dentition for SNP in *TFIP11* (rs134136). Nevertheless, in a recent meta-analysis [62], the T allele of SNP in *TFIP11* (rs134136) was found to be a risk factor. The meta-analysis, however, included patients without distinguishing between permanent and primary dentition, and it is, therefore, not possible to draw any conclusions specific to any type of dentition. The situation is similar for the SNP in *TFIP11* (rs5997096). Our study, as well as others [28, 37, 38, 63], found no association with dental caries. However, Kelly et al. [29] associated this SNP with dental caries of permanent dentition in their meta-analysis. Their work suggested the CT and CC genotypes be protective against tooth decay.

Shaffer et al. [64] performed a meta-analysis comprising six studies, in which they found the SNP in *TUFT1* (rs2337359) to be associated with increased susceptibility to dental caries in permanent dentition. Gerreth et al. [38] reported the A allele and the AA genotype of the SNP in *TUFT1* (rs2337360) to be a risk factor for dental caries in primary dentition in the Polish population. Where SNP in *TUFT1* (rs3790506) is concerned, Patir et al. [52] associated the AG genotype with a higher occurrence of dental caries in the temporary dentition in the Turkish population; Abbasoğlu et al. [28] reported the GG genotype to be protective against dental caries in children with primary dentition. Shimizu et al. [63] reported the A allele of the SNP in *TUFT1* (rs4970957) to be a risk factor for the development of dental caries in the Argentinian and Brazilian populations with mixed dentition. All the 4 above-mentioned SNPs in *TUFT1* were included in this study in the Czech population; neither of them was, however, associated with dental caries.

So far, no functional significance of any of the studied SNPs has been established. All these SNPs are located in intron regions; thus, they possess the ability to affect gene expression and, therefore, the synthesis and/or function of the protein [38]. Several SNPs, for example, rs3796704 in the gene encoding enamelin (*ENAM*) or rs17878486 in *AMELX*, were previously associated with various dental problems, such as amelogenesis imperfecta or molar-incisor hypomineralization [48], despite being in non-coding regions. This strongly supports the hypothesis that even minor alterations in the gene structure could influence protein function, thereby impacting the enamel growth and mineralization and, in effect, altering an individual’s susceptibility to caries. Some SNPs in non-coding regions may be also linked to genetic variants with functional consequences leading to the increased risk of dental caries; nevertheless, testing of the actual influence of non-coding SNPs on protein function and the analysis of linkage disequilibrium throughout the genome remain complicated [63, 65]. However, if an association between an SNP and disease is proved, the SNP can be used as a genetic marker for predicting the risk of a given disease [66].

As shown in Table S1 (Online Resource), the rate of disagreement across studies is high, which only corroborates the opinion that this field has not been sufficiently investigated yet. Dental caries is a multifactorial disease; for this reason, to be able to reach a universally valid conclusion, a study including a large population and a large number of factors affecting the development of dental caries (dietary habits, the level of oral hygiene, presence of fluorides in water or toothpaste, properties of the saliva, oral microbiota, etc.) would be necessary.

Results differ between the primary and permanent dentitions. This was expected (see Table S1), and it was one of the reasons why the associations of gene polymorphisms and dental caries were analyzed in children with primary and permanent dentition separately. Most importantly, the dentitions differ from the morphological perspective and the enamel demineralization is significantly faster in the primary dentition than in the permanent one [4].

Limitations of our study include the low number of subjects in the group of controls with primary dentition; on the other hand, the control groups in some other studies were even lower (see Table S1) (Online Resource). Although we strived to achieve as large population samples as feasible, the control populations (45 patients with primary dentition and 149 with permanent teeth) used for calculation of HWE were likely not large enough as two SNPs (*AMELX* rs946252, rs17878486) were not in HWE. For this reason, the results of these two SNPs must be interpreted with caution. Other factors that have not been considered in this study may also influence the development of dental caries, such as fluoridation or hygiene habits, which may be perceived as another limitation of this study. Still, our study brings a thorough investigation of SNPs in genes encoding proteins involved in enamel formation in relation to dental caries and our extensive literature review is a big contribution to the dental caries research. This study is one of the most complex ones performed so far in the field of genetic predispositions to dental caries. The similarity between MAFs reported for the EUR and CZ populations verifies the validity of our calculations and confirms that our results can be applied on the European Caucasian population in general.

In conclusion, significant associations were found between the occurrence of dental caries, the SNP in *AMELX* (rs17878486), and some SNPs in *KLK4* (rs198968, rs2235091, rs2242670, and rs2978642). These results suggest that gene variants in *AMELX* and *KLK4* may influence the susceptibility to or the severity of dental caries in both primary and permanent dentitions in the Czech population. The literature review also shows that *AMELX* and *KLK4* variabilities have been associated with dental caries more often than variabilities in other genes. Our results contribute to the research of genetic predisposition to dental caries. It is, however, necessary to keep in mind that although we found no associations between SNPs *ALOX15* (rs2619112, rs7217186), *AMELX* (rs946252), *AMBN* (rs34538475), *TFIP11* (rs134136, rs5997096), and *TUFT1* (rs2337359, rs2337360, rs3790506, rs4970957) and dental caries in our study population, we cannot exclude the existence of such associations in other populations.

The presented study is one of the most complex ones so far, separately evaluating the primary and permanent dentitions and evaluating several variants in selected genes (so-called haplotypes). As the functional relevance has not yet been identified for any of the studied SNPs, further research should evaluate the structural and functional significance of these gene variants. Such a study could further clarify the role of polymorphisms in genes encoding proteins involved in the morphology of dental tissues in the etiopathogenesis of dental caries.

## Supplementary Information

Supplement files (Online Resources, MS Word documents) contain a summary of literature review (Table S1), a list of TaqMan® assays used for detection of SNPs (Table S2) and tables with non-significant results (Tables S3-S8).

Below is the link to the electronic supplementary material.

Supplementary file1 (PDF 351 KB)

Supplementary file2 (PDF 153 KB)

Supplementary file3 (PDF 211 KB)

Supplementary file4 (PDF 212 KB)

Supplementary file5 (PDF 164 KB)

Supplementary file6 (PDF 163 KB)

Supplementary file7 (PDF 167 KB)

Supplementary file8 (PDF 184 KB)
