# Supplementary material for: Polymorphisms in genes expressed during amelogenesis and their association with dental caries: a case–control study
Source: Clin Oral Investig. 2022 Nov 24;27(4):1681–95. doi: 10.1007/s00784-022-04794-2 (PMC10102052; doi:10.1007/s00784-022-04794-2)
Supplement: Supplementary file 1 — Supplementary file1 (PDF 351 KB) [file 784_2022_4794_MOESM1_ESM.pdf]

Clinical Oral Investigations

Polymorphisms in genes expressed during amelogenesis and their association with dental caries: a case-control study

Daniela Gachova[1] (ORCID: 0000-0002-5753-0008), Bretislav Lipovy[2] (ORCID: 0000-0001-9187-7606), Tereza Deissova[1] (ORCID: 0000-0003-4853-1233), Lydie Izakovicova Holla[3] (ORCID: 0000-0002-7610-8929), Zdenek Danek[1,4] (ORCID: 0000-0002-0170-2376), Petra Borilova Linhartova[1,3,4,5,*] (ORCID: 0000-0003-0953-3615)

[1] Faculty of Science, RECETOX, Masaryk University, Kotlarska 2, Brno, Czech Republic

[2] Department of Burns and Plastic Surgery, Institution Shared with the University Hospital Brno, Faculty of Medicine, Masaryk University, Jihlavska 20, 62500 Brno, Czech Republic

[3] Clinic of Stomatology, Institution Shared with St. Anne's University Hospital, Faculty of Medicine, Masaryk University, Pekarska 664/53, 60200 Brno, Czech Republic

[4] Clinic of Maxillofacial Surgery, Institution Shared with the University Hospital Brno, Faculty of Medicine, Masaryk University, Jihlavska 20, 62500 Brno, Czech Republic

[5] Department of Pathophysiology, Faculty of Medicine, Masaryk University, Kamenice 5, 62500 Brno, Czech Republic

*Corresponding Author:

Assoc. Prof. Petra Borilova Linhartova, PhD, MBA

Head of the Environmental Genomics Research Group

RECETOX, Faculty of Science, Masaryk University

Kamenice 5

Brno, 625 00, Czech Republic

Tel: +420775393703

E-mail: petra.linhartova@recetox.muni.cz

Table S1. The genetic association case-control studies and meta-analyses focused on polymorphisms in *ALOX15*, *AMBN, AMELX, KLK4, TFIP11 and TUFT1* in patients with dental caries.

Gene	Polymorphism (rs number)	N (dental caries/controls)	Dentition	Population	Association with dental caries	Reference
ALOX15	rs2619112 A/G	100/26	Primary	Czech	NS	CS
		454/147	Permanent	Czech	NS	CS
		106/147	Permanent	Czech	NS	CS
		30/161	Permanent	Mixed	NS	Kelly *et al.* [1]
		267/161	Permanent	Mixed	NS	Kelly *et al.* [1]
		180/161	Permanent	Mixed	NS	Kelly *et al.* [1]
		136/123	Primary	Turkey	NS	Abbasoğlu *et al.* [2]
	rs7217186 C/T	105/45	Primary	Czech	NS	CS
		462/149	Permanent	Czech	NS	CS
		108/149	Permanent	Czech	NS	CS
		48/47	Primary	Poland	NS	Zaorska *et al.* [3]
		30/161	Permanent	Mixed	NS	Kelly *et al.* [1]
		267/161	Permanent	Mixed	NS	Kelly *et al.* [1]
		180/161	Permanent	Mixed	NS	Kelly *et al.* [1]
		136/123	Primary	Turkey	TT genotype (risk)	Abbasoğlu *et al.* [2]
AMBN	rs4694075 C/T	209/224[x] (meta-analysis including 3 studies)	Both	Mixed	NS	Li *et al.* [4]
		30/161	Permanent	Mixed	NS	Kelly *et al.* [1]
		267/161	Permanent	Mixed	NS	Kelly *et al.* [1]
		180/161	Permanent	Mixed	NS	Kelly *et al.* [1]
		48/48	Primary	Poland	NS	Gerreth *et al.* [5]
		136/123	Primary	Turkey	NS	Abbasoğlu *et al.* [2]
		90/82	Primary	Turkey	NS	Shimizu *et al.* [6]
		117/410[x]	Both	Brazil (Curitiba)	NS	Shimizu *et al.* [6]
		298/179[x]	Both	Philippines	C allele (risk)	Shimizu *et al.* [6]
		66/77[x]	Both	Argentina	NS	Shimizu *et al.* [6]
		171/329[x]	Both	Brazil (Rio de Janero)	NS	Shimizu *et al.* [6]
	rs7439186 C/T	889* (meta-analysis including 6 studies)	Both	Mixed	NS	Shaffer *et al.* [7]
		2698* (meta-analysis including 6 studies)	Permanent	Mixed	NS	Shaffer *et al.* [7]
	rs13115627 A/G	382/673 (DMFT≤2)[x]	Permanent	South China	NS	Wang *et al.* [8]
	rs17149026 G/T	889* (meta-analysis including 6 studies)	Both	Mixed	NS	Shaffer *et al.* [7]
		2698* (meta-analysis including 6 studies)	Permanent	Mixed	NS	Shaffer *et al.* [7]
	rs17733915 C/T	889* (meta-analysis including 6 studies)	Both	Mixed	associated**	Shaffer *et al.* [7]
		2698* (meta-analysis including 6 studies)	Permanent	Mixed	NS	Shaffer *et al.* [7]
	rs34538475 G/T	105/45	Primary	Czech	NS	CS
		462/149	Permanent	Czech	NS	CS
		108/149	Permanent	Czech	NS	CS
		143/134[x] (meta-analysis including 2 studies)	Both	Mixed	NS	Li *et al.* [4]
		48/48	Primary	Poland	T allele and TT genotype (protective)	Gerreth *et al.* [5]
		136/123	Primary	Turkey	NS	Abbasoğlu *et al.* [2]
		91/82	Primary	Turkey	T allele (risk)	Patir *et al.* [9]

Gene	Polymorphism (rs number)	N (dental caries/controls)	Dentition	Population	Association with dental caries	Reference
		66/26	Permanent	Guatemala-Maya	NS	Deeley *et al.* [10]
AMELX	rs946252 C/T	100/26	Primary	Czech	NS	CS
		454/147	Permanent	Czech	NS	CS
		106/147	Permanent	Czech	NS	CS
		112/117ˣ (meta-analysis including 2 studies)	Both	Mixed	NS	Li *et al.* [4]
		151/147ˣ (meta-analysis including 3 studies)	Both	Mixed	NS	Sharifi *et al.* [11]
		180/180 (DMFT≤5)ˣ	Permanent	Iran	T alelle (protective)	Koohpeima *et al.* [12]
		136/123	Primary	Turkey	NS	Abbasoğlu *et al.* [2]
		212/146	Both	Mixed	NS	Gasse *et al.* [13]
		90/82	Primary	Turkey	T allele (risk)	Shimizu *et al.* [6]
		298/179ˣ	Both	Philippines	T allele (risk)	Shimizu *et al.* [6]
		117/410ˣ	Both	Brazil (Cutriba)	NS	Shimizu *et al.* [6]
		66/77ˣ	Both	Argentina	NS	Shimizu *et al.* [6]
		171/329ˣ	Both	Brazil (Rio de Janero)	NS	Shimizu *et al.* [6]
	rs2106416 C/T	175/151ˣ (meta-analysis including 3 studies)	Both	Mixed	NS	Li *et al.* [4]
		175/110ˣ (meta-analysis including 2 studies)	Both	Mixed	NS	Sharifi *et al.* [11]
		37/34 (dmft<3)ˣ	Primary	Poland	NS	Olszowski *et al.* [14]
		58/50 (DMFT<3)ˣ	Permanent	Poland	NS	Olszowski *et al.* [14]
	rs5933871 C/T	87/33 (DMFT≤2)ˣ	Permanent	Korea	NS	Kang *et al.* [15]
	rs5934997 C/T	87/33 (DMFT≤2)ˣ	Permanent	Korea	NS	Kang *et al.* [15]
	rs6639060 C/T	157/144ˣ (meta-analysis including 2 studies)	Both	Mixed	NS	Li *et al.* [4]
		157/144ˣ (meta-analysis including 2 studies)	Both	Mixed	NS	Sharifi *et al.* [11]
		77/77 (DMFT≤5)ˣ	Permanent	Turkey	NS	Yildiz *et al.* [16]
	rs17878486 C/T	105/45	Primary	Czech	C allele (risk)	CS
		462/149	Permanent	Czech	T allele and CT + TT genotypes (risk)	CS
		108/149	Permanent	Czech	NS	CS
		249/193ˣ (meta-analysis including 4 studies)	Both	Mixed	C allele (risk) in additive model	Li *et al.* [4]
		249/193ˣ (meta-analysis including 4 studies)	Both	Mixed	NS	Sharifi *et al.* [11]
		48/48	Primary	Poland	T allele and TT genotype (risk)	Gerreth *et al.* [5]
		136/123	Primary	Turkey	NS	Abbasoğlu *et al.* [2]
		87/33 (DMFT≤2)ˣ	Permanent	Korea	NS	Kang *et al.* [15]
		91/82	Primary	Turkey	C allele (risk)	Patir *et al.* [9]
		66/26	Permanent	Guatemala-Maya	T allele (risk)	Deeley *et al.* [10]
	rs184371797 A/C	212/146	Both	Mixed	NS	Gasse *et al.* [13]
	rs200163085 A/G	212/146	Both	Mixed	NS	Gasse *et al.* [13]
KLK4	rs198968 A/G	105/45	Primary	Czech	G allele and GG	CS

Gene	Polymorphism (rs number)	N (dental caries/controls)	Dentition	Population	Association with dental caries	Reference
					genotype vs. AA + AG (risk)	
		462/149	Permanent	Czech	NS	CS
		108/149	Permanent	Czech	NS	CS
		30/161	Permanent	Mixed	NS	Kelly *et al.* [1]
		267/161	Permanent	Mixed	NS	Kelly *et al.* [1]
		180/161	Permanent	Mixed	NS	Kelly *et al.* [1]
		136/123	Primary	Turkey	AG and GG genotypes (protective)	Abbasoğlu *et al.* [2]
	rs198969 C/G	48/48	Primary	Poland	G allele and GG genotype (risk)	Gerreth *et al.* [5]
		82/251	Primary	Mixed	haplotype AC protective (rs2235091/rs198969)	Wang *et al.* [17]
	rs2235091 G/A	105/45	Primary	Czech	NS	CS
		462/149	Permanent	Czech	A allele and A in dominant and additive model (risk)	CS
		108/149	Permanent	Czech	A allele, AA genotype and A in dominant and additive model (risk)	CS
		145/147* (meta-analysis including 2 studies)	Both	Mixed	NS	Li *et al.* [4]
		30/161	Permanent	Mixed	NS	Kelly *et al.* [1]
		267/161	Permanent	Mixed	NS	Kelly *et al.* [1]
		180/161	Permanent	Mixed	NS	Kelly *et al.* [1]
		48/48	Primary	Poland	G allele (risk)	Gerreth *et al.* [5]
		100/100	Permanent	Mixed	NS	Cavallari *et al.* [18]
		136/123	Primary	Turkey	NS	Abbasoğlu *et al.* [2]
		82/251	Primary	Mixed	A allele (protective)	Wang *et al.* [17]
	rs2242670 A/G	105/45	Primary	Czech	GG genotype vs. AA + AG (risk)	CS
		462/149	Permanent	Czech	NS	CS
		108/149	Permanent	Czech	G allele (risk)	CS
		100/100	Permanent	Mixed	AA and AA+AG genotypes (risk)	Cavallari *et al.* [18]
	rs2978642 A/T	105/45	Primary	Czech	NS	CS
		462/149	Permanent	Czech	A allele, AA genotype and A in dominant and additive model (risk)	CS
		108/149	Permanent	Czech	A allele, AA genotype and A in dominant and additive model (risk)	CS
		100/100	Permanent	Mixed	AA + AT genotypes (risk) marginal association	Cavallari *et al.* [18]
	rs2978643 C/G	100/100	Permanent	Mixed	NS	Cavallari *et al.* [18]
TFIP11	rs134134 C/T	889* (meta-analysis including 6 studies)	Primary	Mixed	associated**	Shaffer *et al.* [7]
		2698* (meta-analysis including 6 studies)	Permanent	Mixed	NS	Shaffer *et al.* [7]

Gene	Polymorphism (rs number)	N (dental caries/controls)	Dentition	Population	Association with dental caries	Reference
	rs134135 C/G	889* (meta-analysis including 6 studies)	Primary	Mixed	associated**	Shaffer *et al.* [7]
		2698* (meta-analysis including 6 studies)	Permanent	Mixed	NS	Shaffer *et al.* [7]
	rs134136 C/T	105/45	Primary	Czech	NS	CS
		462/149	Permanent	Czech	NS	CS
		108/149	Permanent	Czech	NS	CS
		220/223ˣ (meta-analysis including 3 studies)	Both	Mixed	NS	Li *et al.* [4]
		48/48	Primary	Poland	NS	Gerreth *et al.* [5]
		136/123	Primary	Turkey	NS	Abbasoğlu *et al.* [2]
		91/82	Primary	Turkey	NS	Patir *et al.* [9]
		66/26	Permanent	Guatemala-Maya	NS	Deeley *et al.* [10]
	rs134143 C/T	382/673 (DMFT≤2)ˣ	Permanent	South China	NS	Wang *et al.* [8]
	rs134145 A/G	889* (meta-analysis including 6 studies)	Primary	Mixed	associated**	Shaffer *et al.* [7]
		2698* (meta-analysis including 6 studies)	Permanent	Mixed	NS	Shaffer *et al.* [7]
	rs713900 A/G	889* (meta-analysis including 6 studies)	Primary	Mixed	NS	Shaffer *et al.* [7]
		2698* (meta-analysis including 6 studies)	Permanent	Mixed	NS	Shaffer *et al.* [7]
	rs2097470 C/T	382/673 (DMFT≤2)ˣ	Permanent	South China	NS	Wang *et al.* [8]
		889* (meta-analysis including 6 studies)	Primary	Mixed	NS	Shaffer *et al.* [7]
		2698* (meta-analysis including 6 studies)	Permanent	Mixed	NS	Shaffer *et al.* [7]
	rs5997096 C/T	105/45	Primary	Czech	NS	CS
		462/149	Permanent	Czech	NS	CS
		108/149	Permanent	Czech	NS	CS
		218/226ˣ (meta-analysis including 3 studies)	Both	Mixed	NS	Li *et al.* [4]
		30/161	Permanent	Mixed	NS	Kelly *et al.* [1]
		267/161	Permanent	Mixed	NS	Kelly *et al.* [1]
		180/161	Permanent	Mixed	CT and CC genotypes (protective)	Kelly *et al.* [1]
		48/48	Primary	Poland	NS	Gerreth *et al.* [5]
		136/123	Primary	Turkey	NS	Abbasoğlu *et al.* [2]
		90/82	Primary	Turkey	NS	Shimizu *et al.* [6]
		298/179ˣ	Both	Philippines	NS	Shimizu *et al.* [6]
		117/410ˣ	Both	Brazil (Curitiba)	NS	Shimizu *et al.* [6]
		66/77ˣ	Both	Argentina	NS	Shimizu *et al.* [6]
		171/329ˣ	Both	Brazil (Rio de Janero)	NS	Shimizu *et al.* [6]
	rs6005060 A/T	889* (meta-analysis including 6 studies)	Primary	Mixed	associated**	Shaffer *et al.* [7]
		2698* (meta-analysis including 6 studies)	Permanent	Mixed	NS	Shaffer *et al.* [7]
	rs17402286 A/G	889* (meta-analysis including 6 studies)	Primary	Mixed	NS	Shaffer *et al.* [7]
		2698* (meta-analysis including 6 studies)	Permanent	Mixed	NS	Shaffer *et al.* [7]

Gene	Polymorphism (rs number)	N (dental caries/controls)	Dentition	Population	Association with dental caries	Reference
TUFT1	rs12749 A/G	889* (meta-analysis including 6 studies)	Primary	Mixed	NS	Shaffer *et al.* [7]
		2698* (meta-analysis including 6 studies)	Permanent	Mixed	NS	Shaffer *et al.* [7]
	rs1045298 C/T	889* (meta-analysis including 6 studies)	Primary	Mixed	NS	Shaffer *et al.* [7]
		2698* (meta-analysis including 6 studies)	Permanent	Mixed	NS	Shaffer *et al.* [7]
	rs2337359 C/T	105/45	Primary	Czech	NS	CS
		462/149	Permanent	Czech	NS	CS
		108/149	Permanent	Czech	NS	CS
		889* (meta-analysis including 6 studies)	Primary	Mixed	NS	Shaffer *et al.* [7]
		2698* (meta-analysis including 6 studies)	Permanent	Mixed	associated**	Shaffer *et al.* [7]
	rs2337360 A/G	105/45	Primary	Czech	NS	CS
		462/149	Permanent	Czech	NS	CS
		108/149	Permanent	Czech	NS	CS
		209/207ˣ (meta-analysis including 3 studies)	Both	Mixed	NS	Li *et al.* [4]
		48/48	Primary	Poland	A allele and AA genotype (risk)	Gerreth *et al.* [5]
		82/251	Primary	Mixed	NS	Wang *et al.* [17]
		91/82	Primary	Turkey	NS	Patir *et al.* [9]
		66/26	Permanent	Guatemala-Maya	NS	Deeley *et al.* [10]
	rs3748608 A/G	82/251	Primary	Mixed	NS	Wang *et al.* [17]
	rs3748609 A/G	82/251	Primary	Mixed	NS	Wang *et al.* [17]
	rs3790506 A/G	105/45	Primary	Czech	NS	CS
		462/149	Permanent	Czech	NS	CS
		108/149	Permanent	Czech	NS	CS
		207/220ˣ (meta-analysis including 3 studies)	Both	Mixed	NS	Li *et al.* [4]
		30/161	Permanent	Mixed	NS	Kelly *et al.* [1]
		267/161	Permanent	Mixed	NS	Kelly *et al.* [1]
		180/161	Permanent	Mixed	NS	Kelly *et al.* [1]
		382/673 (DMFT≤2)ˣ	Permanent	South China	NS	Wang *et al.* [8]
		48/48	Primary	Poland	NS	Gerreth *et al.* [5]
		136/123	Primary	Turkey	GG genotype (protective)	Abbasoğlu *et al.* [2]
		91/82	Primary	Turkey	AG genotype (risk)	Patir *et al.* [9]
		66/26	Permanent	Guatemala-Maya	NS	Deeley *et al.* [10]
	rs3811411 G/T	161/196	Permanent	China	NS	Hu *et al.* [19]
	rs3828054 A/G	136/123	Primary	Turkey	NS	Abbasoğlu *et al.* [2]
		82/251	Primary	Mixed	NS	Wang *et al.* [17]
	rs4970957 A/G	105/45	Primary	Czech	NS	CS
		462/149	Permanent	Czech	NS	CS
		108/149	Permanent	Czech	NS	CS
		221/230ˣ (meta-analysis including 3 studies)	Both	Mixed	NS	Li *et al.* [4]
		48/48	Primary	Poland	NS	Gerreth *et al.* [5]
		136/123	Primary	Turkey	NS	Abbasoğlu *et al.* [2]
		90/82	Primary	Turkey	NS	Shimizu *et al.* [6]

Gene	Polymorphism (rs number)	N (dental caries/controls)	Dentition	Population	Association with dental caries	Reference
		298/179ˣ	Both	Philippines	NS	Shimizu *et al.* [6]
		117/410ˣ	Both	Brazil (Curitiba)	NS	Shimizu *et al.* [6]
		66/77ˣ	Both	Argentina	A allele (risk)	Shimizu *et al.* [6]
		171/329ˣ	Both	Brazil (Rio de Janero)	A allele (risk)	Shimizu *et al.* [6]
	rs6587597 A/G	82/251	Primary	Mixed	NS	Wang *et al.* [17]
	rs7526319 C/T	136/123	Primary	Turkey	NS	Abbasoğlu *et al.* [2]
		82/251	Primary	Mixed	NS	Wang *et al.* [17]
	rs7554707 G/T	82/251	Primary	Mixed	NS	Wang *et al.* [17]
	rs10158855 G/T	889* (meta-analysis including 6 studies)	Primary	Mixed	NS	Shaffer *et al.* [7]
		2698* (meta-analysis including 6 studies)	Permanent	Mixed	NS	Shaffer *et al.* [7]
	rs11204846 A/G	82/251	Primary	Mixed	NS	Wang *et al.* [17]
	rs16833391 C/T	889* (meta-analysis including 6 studies)	Primary	Mixed	NS	Shaffer *et al.* [7]
		2698* (meta-analysis including 6 studies)	Permanent	Mixed	NS	Shaffer *et al.* [7]
	rs17640579 A/G	382/673 (DMFT≤2)ˣ	Permanent	South China	NS	Wang *et al.* [8]
		889* (meta-analysis including 6 studies)	Primary	Mixed	NS	Shaffer *et al.* [7]
		2698* (meta-analysis including 6 studies)	Permanent	Mixed	NS	Shaffer *et al.* [7]

ALOX15, arachidonate 15-lipoxygenase; *AMBN*, ameloblastin; *AMELX*, amelogenin; CS, current study; dmft or DMFT, decay/missing/filled tooth; *KLK4*, kallikrein 4; N, number; NS, nonsignificant; *TFIP11*, tuftelin-interacting protein 11; *TUFT1*, tuftelin 1; for references, see the end of this file.

ˣ high/low caries experience

* study does not specify the number of subjects devided into case/control group

** study does not specify which allele/genotype is risk or protective

REFERENCES

1. Kelly AM, Bezamat M, Modesto A, Vieira AR (2020) Biomarkers for Lifetime Caries-Free Status. J Pers Med 11:23. https://doi.org/10.3390/jpm11010023

2. Abbasoğlu Z, Tanboğa İ, Calvano Küchler E, et al (2015) Early Childhood Caries Is Associated with Genetic Variants in Enamel Formation and Immune Response Genes. Caries Res 49:70–77. https://doi.org/10.1159/000362825

3. Zaorska K, Szczapa T, Borysewicz-Lewicka M, et al (2021) Prediction of Early Childhood Caries Based on Single Nucleotide Polymorphisms Using Neural Networks. Genes 12:462. https://doi.org/10.3390/genes12040462

4. Li X, Liu D, Sun Y, et al (2021) Association of genetic variants in enamel-formation genes with dental caries: A meta- and gene-cluster analysis. Saudi J Biol Sci 28:1645–1653. https://doi.org/10.1016/j.sjbs.2020.11.071

5. Gerreth K, Zaorska K, Zabel M, et al (2017) Chosen single nucleotide polymorphisms (SNPs) of enamel formation genes and dental caries in a population of Polish children. Adv Clin Exp Med 26:899–905. https://doi.org/10.17219/acem/63024

6. Shimizu T, Ho B, Deeley K, et al (2012) Enamel Formation Genes Influence Enamel Microhardness Before and After Cariogenic Challenge. PLoS ONE 7:e45022. https://doi.org/10.1371/journal.pone.0045022

7. Shaffer JR, Carlson JC, Stanley BOC, et al (2015) Effects of enamel matrix genes on dental caries are moderated by fluoride exposures. Hum Genet 134:159–167. https://doi.org/10.1007/s00439-014-1504-7

8. Wang K, Pang L, Tao Y, et al (2020) Association of genetic and environmental factors with dental caries among adolescents in south China: A cross-sectional study. Eur J Paediatr Dent 21:129–136. https://doi.org/10.23804/ejpd.2020.21.02.07

9. Patir A, Seymen F, Yildirim M, et al (2008) Enamel Formation Genes Are Associated with High Caries Experience in Turkish Children. Caries Res 42:394–400. https://doi.org/10.1159/000154785

10. Deeley K, Letra A, Rose EK, et al (2008) Possible Association of *Amelogenin* to High Caries Experience in a Guatemalan-Mayan Population. Caries Res 42:8–13. https://doi.org/10.1159/000111744

11. Sharifi R, Jahedi S, Mozaffari HR, et al (2020) Association of LTF, ENAM, and AMELX polymorphisms with dental caries susceptibility: a meta-analysis. BMC Oral Health 20:132. https://doi.org/10.1186/s12903-020-01121-7

12. Koohpeima F, Derakhshan M, Mokhtari MJ (2019) AMELX Gene Association with Dental Caries in Iranian Adults. Int J Mol Cell Med 8:. https://doi.org/10.22088/IJMCM.BUMS.8.4.294

13. Gasse B, Grabar S, Lafont AG, et al (2013) Common SNPs of *AmelogeninX (AMELX)* and Dental Caries Susceptibility. J Dent Res 92:418–424. https://doi.org/10.1177/0022034513482941

14. Olszowski T, Adler G, Janiszewska-Olszowska J, et al (2012) MBL2, MASP2, AMELX, and ENAM gene polymorphisms and dental caries in Polish children: Genetic polymorphisms and dental caries. Oral Dis 18:389–395. https://doi.org/10.1111/j.1601-0825.2011.01887.x

15. Kang S, Yoon I, Lee H, Cho J (2011) Association between AMELX polymorphisms and dental caries in Koreans: AMELX polymorphisms and dental caries in Koreans. Oral Dis 17:399–406. https://doi.org/10.1111/j.1601-0825.2010.01766.x

16. Yildiz G, Ermis RB, Calapoglu NS, et al (2016) Gene-environment Interactions in the Etiology of Dental Caries. J Dent Res 95:74–79. https://doi.org/10.1177/0022034515605281

17. Wang X, Willing MC, Marazita ML, et al (2012) Genetic and Environmental Factors Associated with Dental Caries in Children: The Iowa Fluoride Study. Caries Res 46:177–184. https://doi.org/10.1159/000337282

18. Cavallari T, Tetu Moyses S, Moyses SJ, Iani Werneck R (2017) KLK4 Gene and Dental Decay: Replication in a South Brazilian Population. Caries Res 51:240–243. https://doi.org/10.1159/000464450

19. Hu X-P, Song T-Z, Zhu Y-Y, et al (2019) Association of *ENAM* , *TUFT1* , *MMP13* , *IL1B* , *IL10 and IL1RN* gene polymorphism and dental caries susceptibility in Chinese children. J Int Med Res 47:1696–1704. https://doi.org/10.1177/0300060519828450
