# Supplementary material for: Polymorphisms in genes expressed during amelogenesis and their association with dental caries: a case–control study
Source: Clin Oral Investig. 2022 Nov 24;27(4):1681–95. doi: 10.1007/s00784-022-04794-2 (PMC10102052; doi:10.1007/s00784-022-04794-2)
Supplement: Supplementary file 2 — Supplementary file2 (PDF 153 KB) [file 784_2022_4794_MOESM2_ESM.pdf]

Clinical Oral Investigations

Polymorphisms in genes expressed during amelogenesis and their association with dental caries: a case-control study

Daniela Gachova[1] (ORCID: 0000-0002-5753-0008), Bretislav Lipovy[2] (ORCID: 0000-0001-9187-7606), Tereza Deissova[1] (ORCID: 0000-0003-4853-1233), Lydie Izakovicova Holla[3] (ORCID: 0000-0002-7610-8929), Zdenek Danek[1,4] (ORCID: 0000-0002-0170-2376), Petra Borilova Linhartova[1,3,4,5,*] (ORCID: 0000-0003-0953-3615)

[1] Faculty of Science, RECETOX, Masaryk University, Kotlarska 2, Brno, Czech Republic

[2] Department of Burns and Plastic Surgery, Institution Shared with the University Hospital Brno, Faculty of Medicine, Masaryk University, Jihlavska 20, 62500 Brno, Czech Republic

[3] Clinic of Stomatology, Institution Shared with St. Anne's University Hospital, Faculty of Medicine, Masaryk University, Pekarska 664/53, 60200 Brno, Czech Republic

[4] Clinic of Maxillofacial Surgery, Institution Shared with the University Hospital Brno, Faculty of Medicine, Masaryk University, Jihlavska 20, 62500 Brno, Czech Republic

[5] Department of Pathophysiology, Faculty of Medicine, Masaryk University, Kamenice 5, 62500 Brno, Czech Republic

*Corresponding Author:

Assoc. Prof. Petra Borilova Linhartova, PhD, MBA

Head of the Environmental Genomics Research Group

RECETOX, Faculty of Science, Masaryk University

Kamenice 5

Brno, 625 00, Czech Republic

Tel: +420775393703

E-mail: petra.linhartova@recetox.muni.cz

Table S2. TaqMan® assays for genotyping of 15 selected single nucleotide polymorphisms.

**gene**	**SNP**	**assay identification number** (ThermoScientific, Waltham, USA)
ALOX15	rs2619112	C_3109408_10_A/G_VIC/FAM
	rs7217186	C_27844482_20_C/T_VIC/FAM
AMBN	rs34538475	C_496502_10_G/T_VIC/FAM
AMELX	rs946252	C_9527787_20_C/T_VIC/FAM
	rs17878486	C_2190967_10_C/T_VIC/FAM
KLK4	rs198968	C_736068_1_A/G_VIC/FAM
	rs2235091	C_15879128_10_G/A_ VIC/FAM
	rs2242670	C_16057276_10_A/G_VIC/FAM
	rs2978642	C_15879137_10_A/T_VIC/FAM
TFIP11	rs134136	C_2225865_1_C/T_VIC/FAM
	rs5997096	C_29903745_10_C/T_VIC/FAM
TUFT1	rs2337359	C_40820_10_C/T_VIC/FAM
	rs2337360	C_26043576_10_A/G_VIC/FAM
	rs3790506	C_34467_10_A/G_VIC/FAM
	rs4970957	C_27907011_10_A/G_VIC/FAM

ALOX15, arachidonate 15-lipoxygenase; *AMBN*, ameloblastin; *AMELX*, amelogenin; FAM, FAM™ fluorescent dye; KLK4, kallikrein 4; SNP, single nucleotide polymorphism; *TFIP11*, tuftelin-interacting protein 11; *TUFT1*, tuftelin 1; VIC, VIC® fluorescent dye
