# Supplementary material for: Polymorphisms in genes expressed during amelogenesis and their association with dental caries: a case–control study
Source: Clin Oral Investig. 2022 Nov 24;27(4):1681–95. doi: 10.1007/s00784-022-04794-2 (PMC10102052; doi:10.1007/s00784-022-04794-2)
Supplement: Supplementary file 3 — Supplementary file3 (PDF 211 KB) [file 784_2022_4794_MOESM3_ESM.pdf]

Clinical Oral Investigations

Polymorphisms in genes expressed during amelogenesis and their association with dental caries: a case-control study

Daniela Gachova[1] (ORCID: 0000-0002-5753-0008), Bretislav Lipovy[2] (ORCID: 0000-0001-9187-7606), Tereza Deissova[1] (ORCID: 0000-0003-4853-1233), Lydie Izakovicova Holla[3] (ORCID: 0000-0002-7610-8929), Zdenek Danek[1,4] (ORCID: 0000-0002-0170-2376), Petra Borilova Linhartova[1,3,4,5,*] (ORCID: 0000-0003-0953-3615)

[1] Faculty of Science, RECETOX, Masaryk University, Kotlarska 2, Brno, Czech Republic

[2] Department of Burns and Plastic Surgery, Institution Shared with the University Hospital Brno, Faculty of Medicine, Masaryk University, Jihlavska 20, 62500 Brno, Czech Republic

[3] Clinic of Stomatology, Institution Shared with St. Anne's University Hospital, Faculty of Medicine, Masaryk University, Pekarska 664/53, 60200 Brno, Czech Republic

[4] Clinic of Maxillofacial Surgery, Institution Shared with the University Hospital Brno, Faculty of Medicine, Masaryk University, Jihlavska 20, 62500 Brno, Czech Republic

[5] Department of Pathophysiology, Faculty of Medicine, Masaryk University, Kamenice 5, 62500 Brno, Czech Republic

*Corresponding Author:

Assoc. Prof. Petra Borilova Linhartova, PhD, MBA

Head of the Environmental Genomics Research Group

RECETOX, Faculty of Science, Masaryk University

Kamenice 5

Brno, 625 00, Czech Republic

Tel: +420775393703

E-mail: petra.linhartova@recetox.muni.cz

Table S3. Allele and genotype frequencies of selected single nucleotide polymorphisms (SNPs) in genes encoding arachidonate 15-lipoxygenase (*ALOX15*) or ameloblastin (*AMBN*) for dental caries in children with primary dentition and with permanent dentition.

gene *ALOX15* SNP		Primary dmft = 0 N = 45 (%)	Primary dmft ≥ 10 N = 105 (%)	p-value	Permanent DMFT = 0 N = 149 (%)	Permanent DMFT > 0 N = 462 (%)	p-value	Permanent DMFT ≥ 6 N = 108 (%)	p-value
rs2619112♦	A	28 (53.8)	100 (50.0)	0.368	142 (48.3)	440 (48.5)	0.508	101 (47.6)	0.478
	G	24 (46.2)	100 (50.0)		152 (51.7)	468 (51.5)		111 (52.4)	
	AA	7 (26.9)	27 (27.0)		34 (23.1)	107 (23.5)		22 (20.8)	
	AG	14 (53.8)	46 (46.0)	0.685	74 (50.3)	226 (49.8)	0.992	57 (53.8)	0.851
	GG	5 (19,2)	27 (27.0)		39 (26.5)	121 (26.7)		27 (25.5)	
rs7217186	C	49 (54.4)	110 (52.4)	0.420	141 (47.3)	454 (49.1)	0.316	105 (48.6)	0.420
	T	41 (45.6)	100 (47.6)		157 (52.7)	470 (50.9)		111 (51.4)	
	CC	13 (28.9)	30 (28.6)		31 (20.8)	114 (24.7)		25 (23.1)	
	CT	23 (51.1)	50 (47.6)	0.869	79 (53.0)	226 (48.9)	0.579	55 (50.9)	0.899
	TT	9 (20.0)	25 (23.8)		39 (26.2)	122 (26.4)		28 (25.9)	

gene *AMBN* SNP		Primary dmft = 0 N = 45 (%)	Primary dmft ≥ 10 N = 105 (%)	p-value	Permanent DMFT = 0 N = 149 (%)	Permanent DMFT > 0 N = 462 (%)	p-value	Permanent DMFT ≥ 6 N = 108 (%)	p-value
rs34538475	T	18 (20.0)	60 (28.6)	0.078	82 (27.5)	238 (25.8)	0.298	67 (31.0)	0.222
	G	72 (80.0)	150 (71.4)		216 (72.5)	686 (74.2)		149 (69.0)	
	TT	2 (4.4)	12 (11.4)		12 (8.1)	34 (7.4)		13 (12.0)	
	GT	14 (31.1)	36 (34.3)	0.315	58 (38.9)	170 (36.8)	0.830	41 (38.0)	0.564
	GG	29 (64.4)	57 (54.3)		79 (53.0)	258 (55.8)		54 (50.0)	
	TT + GT	16 (35.6)	48 (45.7)	0.165	70 (47.0)	204 (44.2)	0.305	54 (50.0)	0.362
	GT + GG	43 (95.6)	93 (88.6)	0.148	137 (91.9)	428 (92.6)	0.450	95 (88.0)	0.197

dmft or DMFT, decay/missing/filled tooth; N (%), values represent numbers (%) of subjects

♦ 5 subjects in the group with primary dentition with dmft ≥ 10 and 19 subjects with dmft = 0 were not analysed, 8 subjects in the group with permanent dentition with DMFT > 0, 2 subjects with DMFT ≥ 6 and 2 subjects with DMFT = 0 were not analysed

Differences in allele frequencies were tested by the Fisher-exact test.
