# Supplementary material for: Polymorphisms in genes expressed during amelogenesis and their association with dental caries: a case–control study
Source: Clin Oral Investig. 2022 Nov 24;27(4):1681–95. doi: 10.1007/s00784-022-04794-2 (PMC10102052; doi:10.1007/s00784-022-04794-2)
Supplement: Supplementary file 4 — Supplementary file4 (PDF 212 KB) [file 784_2022_4794_MOESM4_ESM.pdf]

Clinical Oral Investigations

Polymorphisms in genes expressed during amelogenesis and their association with dental caries: a case-control study

Daniela Gachova[1] (ORCID: 0000-0002-5753-0008), Bretislav Lipovy[2] (ORCID: 0000-0001-9187-7606), Tereza Deissova[1] (ORCID: 0000-0003-4853-1233), Lydie Izakovicova Holla[3] (ORCID: 0000-0002-7610-8929), Zdenek Danek[1,4] (ORCID: 0000-0002-0170-2376), Petra Borilova Linhartova[1,3,4,5,*] (ORCID: 0000-0003-0953-3615)

[1] Faculty of Science, RECETOX, Masaryk University, Kotlarska 2, Brno, Czech Republic

[2] Department of Burns and Plastic Surgery, Institution Shared with the University Hospital Brno, Faculty of Medicine, Masaryk University, Jihlavska 20, 62500 Brno, Czech Republic

[3] Clinic of Stomatology, Institution Shared with St. Anne's University Hospital, Faculty of Medicine, Masaryk University, Pekarska 664/53, 60200 Brno, Czech Republic

[4] Clinic of Maxillofacial Surgery, Institution Shared with the University Hospital Brno, Faculty of Medicine, Masaryk University, Jihlavska 20, 62500 Brno, Czech Republic

[5] Department of Pathophysiology, Faculty of Medicine, Masaryk University, Kamenice 5, 62500 Brno, Czech Republic

*Corresponding Author:

Assoc. Prof. Petra Borilova Linhartova, PhD, MBA

Head of the Environmental Genomics Research Group

RECETOX, Faculty of Science, Masaryk University

Kamenice 5

Brno, 625 00, Czech Republic

Tel: +420775393703

E-mail: petra.linhartova@recetox.muni.cz

Table S4. Allele and genotype frequencies of selected SNPs in genes encoding tuftelin-interacting protein 11 (*TFIP11*) and tuftelin 1 (*TUFT1*) for dental caries in children with primary dentition and with permanent dentition.

**gene *TFIP11* SNP**		**Primary dmft = 0 N = 45 (%)**	**Primary dmft ≥ 10 N = 105 (%)**	**p-value**	**Permanent DMFT = 0 N = 149 (%)**	**Permanent DMFT > 0 N = 462 (%)**	**p-value**	**Permanent DMFT ≥ 6 N = 108 (%)**	**p-value**
rs134136	T	41 (45.6)	85 (40.5)	0.245	120 (40.3)	354 (38.3)	0.296	78 (36.1)	0.194
	C	49 (54.4)	125 (59.5)		178 (59.7)	570 (61.7)		138 (63.9)	
	TT	9 (20.0)	18 (17.1)	0.682	25 (16.8)	69 (14.9)	0.828	14 (13.0)	0.625
	CT	23 (51.1)	49 (46.7)		70 (47.0)	216 (46.8)		50 (46.3)	
	CC	13 (28.9)	38 (36.2)		54 (36.2)	177 (38.3)		44 (40.7)	
rs5997096	T	45 (50.0)	103 (49.0)	0.490	147 (49.3)	429 (46.4)	0.210	95 (44.0)	0.134
	C	45 (50.0)	107 (51.0)		151 (50.7)	495 (53.6)		121 (56.0)	
	TT	10 (22.2)	28 (26.7)	0.476	35 (23.5)	101 (21.9)	0.612	20 (18.5)	0.476
	CT	25 (55.6)	47 (44.8)		77 (51.7)	227 (49.1)		55 (50.9)	
	CC	10 (22.2)	30 (28.6)		37 (24.8)	134 (29.0)		33 (30.6)	

**gene *TUFT1* SNP**		**Primary dmft = 0 N = 45 (%)**	**Primary dmft ≥ 10 N = 105 (%)**	**p-value**	**Permanent DMFT = 0 N = 149 (%)**	**Permanent DMFT > 0 N = 462 (%)**	**p-value**	**Permanent DMFT ≥ 6 N = 108 (%)**	**p-value**
rs2337359	C	27 (30.0)	52 (24.8)	0.211	56 (18.8)	195 (21.1)	0.220	41 (19.0)	0.522
	T	63 (70.0)	158 (75.2)		242 (81.2)	729 (78.9)		175 (81.0)	
	CC	4 (8.9)	3 (2.9)	0.273	3 (2.0)	24 (5.2)	0.256	3 (2.8)	0.912
	CT	19 (42.2)	46 (43.8)		50 (33.6)	147 (31.8)		35 (32.4)	
	TT	22 (48.9)	56 (53.3)		96 (64.4)	291 (63.0)		70 (64.8)	
	CC + CT	23 (51.1)	49 (46.7)	0.374	53 (35.6)	171 (37.0)	0.415	38 (35.2)	0.528
	CT + TT	41 (91.1)	102 (97.1)	0.121	146 (98.0)	438 (94.8)	0.072	105 (97.2)	0.498
rs2337360	A	33 (36.7)	93 (44.3)	0.136	138 (46.3)	386 (41.8)	0.096	92 (42.6)	0.228
	G	57 (63.3)	117 (55.7)		160 (53.7)	538 (58.2)		124 (57.4)	
	AA	8 (17.8)	22 (21.0)	0.368	30 (20.1)	84 (18.2)	0.275	20 (18.5)	0.604
	AG	17 (37.8)	49 (46.7)		78 (52.3)	218 (47.2)		52 (48.1)	
	GG	20 (44.4)	34 (32.4)		41 (27.5)	160 (34.6)		36 (33.3)	
	AA + AG	25 (55.6)	71 (67.6)	0.111	108 (72.5)	302 (65.4)	0.065	72 (66.7)	0.193
rs3790506	A	24 (26.7)	68 (32.4)	0.199	88 (29.5)	263 (28.5)	0.388	60 (27.8)	0.370
	G	66 (73.3)	142 (67.6)		210 (70.5)	661 (71.5)		156 (72.2)	
	AA	6 (13.3)	13 (12.4)	0.284	13 (8.7)	31 (6.7)	0.694	8 (7.4)	0.903

gene *TUFT1* SNP		Primary dmft = 0 N = 45 (%)	Primary dmft ≥ 10 N = 105 (%)	p-value	Permanent DMFT = 0 N = 149 (%)	Permanent DMFT > 0 N = 462 (%)	p-value	Permanent DMFT ≥ 6 N = 108 (%)	p-value
	AG	12 (26.7)	42 (40.0)		62 (41.6)	201 (43.5)		44 (40.7)	
	GG	27 (60.0)	50 (47.6)		74 (49.7)	230 (49.8)		56 (51.9)	
rs4970957	G	15 (16.7)	32 (15.2)	0.439	48 (16.1)	183 (19.8)	0.090	39 (18.1)	0.321
	A	75 (83.3)	178 (84.8)		250 (83.9)	741 (80.2)		177 (81.9)	
	GG	0 (0.0)	2 (1.9)		4 (2.7)	22 (4.8)		5 (4.6)	
	AG	15 (33.3)	28 (26.7)	0.485	40 (26.8)	139 (30.1)	0.363	29 (26.9)	0.701
	AA	30 (66.7)	75 (71.4)		105 (70.5)	301 (65.2)		74 (68.5)	

dmft or DMFT, decay/missing/filled tooth; N (%), values represent numbers (%) of subjects; SNP, single nucleotide polymorphism
