# Supplementary material for: Polymorphisms in genes expressed during amelogenesis and their association with dental caries: a case–control study
Source: Clin Oral Investig. 2022 Nov 24;27(4):1681–95. doi: 10.1007/s00784-022-04794-2 (PMC10102052; doi:10.1007/s00784-022-04794-2)
Supplement: Supplementary file 5 — Supplementary file5 (PDF 164 KB) [file 784_2022_4794_MOESM5_ESM.pdf]

Clinical Oral Investigations

Polymorphisms in genes expressed during amelogenesis and their association with dental caries: a case-control study

Daniela Gachova[1] (ORCID: 0000-0002-5753-0008), Bretislav Lipovy[2] (ORCID: 0000-0001-9187-7606), Tereza Deissova[1] (ORCID: 0000-0003-4853-1233), Lydie Izakovicova Holla[3] (ORCID: 0000-0002-7610-8929), Zdenek Danek[1,4] (ORCID: 0000-0002-0170-2376), Petra Borilova Linhartova[1,3,4,5,*] (ORCID: 0000-0003-0953-3615)

[1] Faculty of Science, RECETOX, Masaryk University, Kotlarska 2, Brno, Czech Republic

[2] Department of Burns and Plastic Surgery, Institution Shared with the University Hospital Brno, Faculty of Medicine, Masaryk University, Jihlavska 20, 62500 Brno, Czech Republic

[3] Clinic of Stomatology, Institution Shared with St. Anne's University Hospital, Faculty of Medicine, Masaryk University, Pekarska 664/53, 60200 Brno, Czech Republic

[4] Clinic of Maxillofacial Surgery, Institution Shared with the University Hospital Brno, Faculty of Medicine, Masaryk University, Jihlavska 20, 62500 Brno, Czech Republic

[5] Department of Pathophysiology, Faculty of Medicine, Masaryk University, Kamenice 5, 62500 Brno, Czech Republic

*Corresponding Author:

Assoc. Prof. Petra Borilova Linhartova, PhD, MBA

Head of the Environmental Genomics Research Group

RECETOX, Faculty of Science, Masaryk University

Kamenice 5

Brno, 625 00, Czech Republic

Tel: +420775393703

E-mail: petra.linhartova@recetox.muni.cz

Table S5. Haplotype analysis of single nucleotide polymorphisms (SNPs) in the gene encoding arachidonate 15-lipoxygenase (*ALOX15*) and its association with dental caries in the primary dentition with dmft ≥ 10 and permanent dentition with DMFT > 0 and DMFT ≥ 6.

rs2619112	rs7217186	Primary dmft = 0	Primary dmft ≥ 10	OR	CI	p-value	Permanent DMFT = 0	Permanent DMFT > 0	OR	CI	p-value	Permanent DMFT ≥ 6	OR	CI	p-value
A	C	51.8 %	50.0 %	0.908	0.493-1.671	0.756	43.1 %	45.9 %	1.163	0.892-1.515	0.265	45.0 %	1.121	0.786-1.598	0.529
G	T	40.3 %	45.6 %	1.309	0.704-2.433	0.392	49.5 %	48.9 %	0.966	0.742-1.256	0.796	49.3 %	1.042	0.732-1.483	0.821
G	C	5.9 %	3.6 %	0.505	0.122-2.092	0.366	2.9 %	2.6 %	0.927	0.410-2.095	0.856	2.7 %	0.864	0.279-2.677	0.798
A	T	2.1 %	0.8 %	0.256	0.016-4.168	0.355	4.6 %	2.5 %	0.387	0.184-0.814	**0.015**[x]	3.0 %	0.206	0.046-0.922	**0.015**[x]

CI, confidence interval; dmft or DMFT, decay/missing/filled tooth; OR, odds ratio

[x]Not significant after the Bonferroni correction.

Haplotypes are ordered according to decreasing haplotype frequency in the healthy controls from the group with primary dentition.
