# Supplementary material for: Polymorphisms in genes expressed during amelogenesis and their association with dental caries: a case–control study
Source: Clin Oral Investig. 2022 Nov 24;27(4):1681–95. doi: 10.1007/s00784-022-04794-2 (PMC10102052; doi:10.1007/s00784-022-04794-2)
Supplement: Supplementary file 6 — Supplementary file6 (PDF 163 KB) [file 784_2022_4794_MOESM6_ESM.pdf]

Clinical Oral Investigations

Polymorphisms in genes expressed during amelogenesis and their association with dental caries: a case-control study

Daniela Gachova[1] (ORCID: 0000-0002-5753-0008), Bretislav Lipovy[2] (ORCID: 0000-0001-9187-7606), Tereza Deissova[1] (ORCID: 0000-0003-4853-1233), Lydie Izakovicova Holla[3] (ORCID: 0000-0002-7610-8929), Zdenek Danek[1,4] (ORCID: 0000-0002-0170-2376), Petra Borilova Linhartova[1,3,4,5,*] (ORCID: 0000-0003-0953-3615)

[1] Faculty of Science, RECETOX, Masaryk University, Kotlarska 2, Brno, Czech Republic

[2] Department of Burns and Plastic Surgery, Institution Shared with the University Hospital Brno, Faculty of Medicine, Masaryk University, Jihlavska 20, 62500 Brno, Czech Republic

[3] Clinic of Stomatology, Institution Shared with St. Anne's University Hospital, Faculty of Medicine, Masaryk University, Pekarska 664/53, 60200 Brno, Czech Republic

[4] Clinic of Maxillofacial Surgery, Institution Shared with the University Hospital Brno, Faculty of Medicine, Masaryk University, Jihlavska 20, 62500 Brno, Czech Republic

[5] Department of Pathophysiology, Faculty of Medicine, Masaryk University, Kamenice 5, 62500 Brno, Czech Republic

*Corresponding Author:

Assoc. Prof. Petra Borilova Linhartova, PhD, MBA

Head of the Environmental Genomics Research Group

RECETOX, Faculty of Science, Masaryk University

Kamenice 5

Brno, 625 00, Czech Republic

Tel: +420775393703

E-mail: petra.linhartova@recetox.muni.cz

Table S6. Haplotype analysis of single nucleotide polymorphisms (SNPs) in gene encoding amelogenin (*AMELX)* and their association with dental caries in the primary dentition with dmft ≥ 10 and permanent dentition with DMFT > 0 and DMFT ≥ 6.

rs946252	rs17878486	Primary dmft = 0	Primary dmft ≥ 10	OR	CI	p-value	Permanent DMFT = 0	Permanent DMFT > 0	OR	CI	p-value	Permanent DMFT ≥ 6	OR	CI	p-value
C	T	51.9 %	50.7 %	0.908	0.493-1.671	0.756	47.6 %	49.7 %	1.130	0.868-1.470	0.363	49.2 %	1.142	0.802-1.627	0.460
T	T	30.8 %	21.9 %	0.580	0.293-1.147	0.125	23.2 %	25.9 %	1.198	0.881-1.629	0.244	23.7 %	1.210	0.802-1.826	0.364
C	C	17.3 %	25.1 %	1.858	0.850-4.061	0.105	27.2 %	23.5 %	0.757	0.561-1.022	0.072	25.9 %	0.764	0.508-1.149	0.194
T	C	0.0 %	2.3 %	0.000	0.000-0.000	-	2.0 %	0.9 %	0.256	0.068-0.959	**0.046**[x]	1.2 %	0.000	0.000-0.000	-

CI, confidence interval; dmft or DMFT, decay/missing/filled tooth; OR, odds ratio

[x]Not significant after the Bonferroni approach.

Haplotypes are ordered according to decreasing haplotype frequency in the healthy controls from the group with primary dentition.
