# Supplementary material for: Polymorphisms in genes expressed during amelogenesis and their association with dental caries: a case–control study
Source: Clin Oral Investig. 2022 Nov 24;27(4):1681–95. doi: 10.1007/s00784-022-04794-2 (PMC10102052; doi:10.1007/s00784-022-04794-2)
Supplement: Supplementary file 7 — Supplementary file7 (PDF 167 KB) [file 784_2022_4794_MOESM7_ESM.pdf]

Clinical Oral Investigations

Polymorphisms in genes expressed during amelogenesis and their association with dental caries: a case-control study

Daniela Gachova[1] (ORCID: 0000-0002-5753-0008), Bretislav Lipovy[2] (ORCID: 0000-0001-9187-7606), Tereza Deissova[1] (ORCID: 0000-0003-4853-1233), Lydie Izakovicova Holla[3] (ORCID: 0000-0002-7610-8929), Zdenek Danek[1,4] (ORCID: 0000-0002-0170-2376), Petra Borilova Linhartova[1,3,4,5,*] (ORCID: 0000-0003-0953-3615)

[1] Faculty of Science, RECETOX, Masaryk University, Kotlarska 2, Brno, Czech Republic

[2] Department of Burns and Plastic Surgery, Institution Shared with the University Hospital Brno, Faculty of Medicine, Masaryk University, Jihlavska 20, 62500 Brno, Czech Republic

[3] Clinic of Stomatology, Institution Shared with St. Anne's University Hospital, Faculty of Medicine, Masaryk University, Pekarska 664/53, 60200 Brno, Czech Republic

[4] Clinic of Maxillofacial Surgery, Institution Shared with the University Hospital Brno, Faculty of Medicine, Masaryk University, Jihlavska 20, 62500 Brno, Czech Republic

[5] Department of Pathophysiology, Faculty of Medicine, Masaryk University, Kamenice 5, 62500 Brno, Czech Republic

*Corresponding Author:

Assoc. Prof. Petra Borilova Linhartova, PhD, MBA

Head of the Environmental Genomics Research Group

RECETOX, Faculty of Science, Masaryk University

Kamenice 5

Brno, 625 00, Czech Republic

Tel: +420775393703

E-mail: petra.linhartova@recetox.muni.cz

Table S7. Haplotype analysis of single nucleotide polymorphisms (SNPs) in the gene encoding tuftelin-interacting protein 11 (*TFIP11)* and its association with dental caries in primary dentition with dmft ≥ 10 and permanent dentition with DMFT > 0 and DMFT ≥ 6.

rs134136	rs5997096	Primary dmft = 0 N = 45 (%)	Primary dmft ≥ 10 N = 105 (%)	OR	CI	p-value	Permanent DMFT = 0 N = 149 (%)	Permanent DMFT > 0 N = 462 (%)	OR	CI	p-value	Permanent DMFT ≥ 6 N = 108 (%)	OR	CI	p-value
C	C	50.0 %	50.7 %	1.039	0.634-1.702	0.880	50.7 %	52.8 %	1.123	0.865-1.459	0.383	52.9 %	1.240	0.872-1.763	0.230
T	T	45.6 %	42.0 %	0.813	0.494-1.337	0.415	40.3 %	38.4 %	0.904	0.693-1.181	0.462	38.5 %	0.838	0.584-1.204	0.338
C	T	4.4 %	7.3 %	2.016	0.662-6.134	0.189	9.1 %	8.5 %	0.912	0.576-1.444	0.698	8.6 %	0.857	0.455-1.616	0.633
T	C	-	-	-	-	-	0.0 %	0.2 %	0.000	0.000-0.000	-	-	-	-	-

CI, confidence interval; dmft or DMFT, decay/missing/filled tooth; OR, odds ratio

Haplotypes are ordered according to decreasing haplotype frequency in the healthy controls from the group with primary dentition.
