# Supplementary material for: Polymorphisms in genes expressed during amelogenesis and their association with dental caries: a case–control study
Source: Clin Oral Investig. 2022 Nov 24;27(4):1681–95. doi: 10.1007/s00784-022-04794-2 (PMC10102052; doi:10.1007/s00784-022-04794-2)
Supplement: Supplementary file 8 — Supplementary file8 (PDF 184 KB) [file 784_2022_4794_MOESM8_ESM.pdf]

Clinical Oral Investigations

Polymorphisms in genes expressed during amelogenesis and their association with dental caries: a case-control study

Daniela Gachova[1] (ORCID: 0000-0002-5753-0008), Bretislav Lipovy[2] (ORCID: 0000-0001-9187-7606), Tereza Deissova[1] (ORCID: 0000-0003-4853-1233), Lydie Izakovicova Holla[3] (ORCID: 0000-0002-7610-8929), Zdenek Danek[1,4] (ORCID: 0000-0002-0170-2376), Petra Borilova Linhartova[1,3,4,5,*] (ORCID: 0000-0003-0953-3615)

[1] Faculty of Science, RECETOX, Masaryk University, Kotlarska 2, Brno, Czech Republic

[2] Department of Burns and Plastic Surgery, Institution Shared with the University Hospital Brno, Faculty of Medicine, Masaryk University, Jihlavska 20, 62500 Brno, Czech Republic

[3] Clinic of Stomatology, Institution Shared with St. Anne's University Hospital, Faculty of Medicine, Masaryk University, Pekarska 664/53, 60200 Brno, Czech Republic

[4] Clinic of Maxillofacial Surgery, Institution Shared with the University Hospital Brno, Faculty of Medicine, Masaryk University, Jihlavska 20, 62500 Brno, Czech Republic

[5] Department of Pathophysiology, Faculty of Medicine, Masaryk University, Kamenice 5, 62500 Brno, Czech Republic

*Corresponding Author:

Assoc. Prof. Petra Borilova Linhartova, PhD, MBA

Head of the Environmental Genomics Research Group

RECETOX, Faculty of Science, Masaryk University

Kamenice 5

Brno, 625 00, Czech Republic

Tel: +420775393703

E-mail: petra.linhartova@recetox.muni.cz

Table S8. Haplotype analysis of single nucleotide polymorphisms (SNPs) in gene encoding tuftelin 1 (*TUFT1)* and its association with dental caries in primary dentition with dmft ≥ 10 and permanent dentition with DMFT > 0 and DMFT ≥ 6.

rs3790506	rs2337359	rs2337360	rs4970957	Primary dmft = 0 N = 45 (%)	Primary dmft ≥ 10 N = 105 (%)	OR	CI	p-value	Permanent DMFT = 0 N = 149 (%)	Permanent DMFT > 0 N = 462 (%)	OR	CI	p-value	Permanent DMFT ≥ 6 N = 108 (%)	OR	CI	p-value
G	T	G	A	27.2 %	24.3 %	0.846	0.468-1.530	0.582	25.6 %	23.3 %	0.878	0.644-1.196	0.410	24.5 %	0.921	0.609-1.394	0.697
A	T	A	A	20.1 %	21.3 %	1.250	0.682-2.292	0.466	26.3 %	21.4 %	0.817	0.603-1.107	0.196	22.6 %	0.737	0.487-1.116	0.147
G	C	G	A	19.2 %	13.7 %	0.623	0.329-1.178	0.150	10.9 %	11.8 %	1.149	0.774-1.707	0.487	11.3 %	1.174	0.698-1.974	0.546
G	T	G	G	15.1 %	13.3 %	0.833	0.424-1.638	0.600	14.7 %	15.2 %	1.131	0.783-1.634	0.508	15.1 %	1.059	0.656-1.711	0.814
G	T	A	A	8.2 %	13.2 %	1.559	0.709-3.425	0.255	14.3 %	16.7 %	0.999	0.706-1.413	0.995	16.6 %	1.307	0.813-2.099	0.270
A	C	A	A	4.4 %	3.6 %	0.524	0.137-2.000	0.353	0.9 %	2.5 %	1.077	0.428-2.707	0.874	2.5 %	3.507	0.674-18.249	0.113
G	C	A	A	3.9 %	4.1 %	1.517	0.309-7.450	0.595	4.9 %	1.9 %	0.191	0.045-0.804	**0.022**[x]	3.0 %	0.205	0.046-0.918	**0.014**[x]
A	C	G	A	2.0 %	3.2 %	1.970	0.417-9.306	0.361	1.1 %	1.8 %	1.543	0.521-4.572	0.413	1.2 %	1.035	0.229-4.674	0.964
A	T	G	A	0.0 %	1.5 %	0.000	0.000-0.000	-	0.0 %	1.8 %	4.569	0.598-34.896	0.068	1.4 %	0.000	0.000-0.000	-
G	C	G	G	0.0 %	1.2 %	0.000	0.000-0.000	-	0.0 %	2.4 %	2.212	0.768-6.375	0.107	0.6 %	0.000	0.000-0.000	-
A	T	G	G	0.0 %	0.6 %	0.000	0.000-0.000	-	0.4 %	0.8 %	0.000	0.000-0.000	-	0.9 %	4.183	0.432-40.492	0.179
A	C	G	G	-	-	-	-	-	0.8 %	0.3 %	0.322	0.020-5.161	0.435	0.3 %	0.000	0.000-0.000	-

CI, confidence interval; dmft or DMFT, decay/missing/filled tooth; OR, odds ratio

[x]Not significant after the Bonferroni approach.

Haplotypes are ordered according to decreasing haplotype frequency in the healthy controls from the group with primary dentition.
